# InterVelo: a mutually enhancing model for estimating pseudotime and RNA velocity in multi-omic single-cell data

**DOI:** 10.1093/bioinformatics/btaf500

**Published:** 2025-09-10

**Authors:** Yurou Wang, Zhixiang Lin, Tao Wang

**Affiliations:** Department of Bioinformatics and Biostatistics, School of Life Sciences and Biotechnology, Shanghai Jiao Tong University, 200240 Shanghai, China; SJTU-Yale Joint Center for Biostatistics and Data Science, Technical Center for Digital Medicine, National Center for Translational Medicine, Shanghai Jiao Tong University, 200240 Shanghai, China; Department of Statistics, The Chinese University of Hong Kong, 999077 Shatin, Hong Kong SAR, China; Department of Bioinformatics and Biostatistics, School of Life Sciences and Biotechnology, Shanghai Jiao Tong University, 200240 Shanghai, China; SJTU-Yale Joint Center for Biostatistics and Data Science, Technical Center for Digital Medicine, National Center for Translational Medicine, Shanghai Jiao Tong University, 200240 Shanghai, China; Department of Statistics and MOE-LSC and CMA-Shanghai, School of Mathematical Sciences, Shanghai Jiao Tong University, 200240 Shanghai, China; MoE Key Lab of Artificial Intelligence, AI Institute, Shanghai Jiao Tong University, 200240 Shanghai, China

## Abstract

**Motivation:**

RNA velocity has become a powerful tool for uncovering transcriptional dynamics in snapshot single-cell data. However, current RNA velocity approaches often assume constant transcriptional rates and treat genes independently with gene-specific times, which may introduce biases and deviate from biological realities. Here, we present InterVelo, a novel deep learning framework that simultaneously learns cellular pseudotime and RNA velocity.

**Results:**

InterVelo leverages an unsupervised cellular time to guide RNA velocity estimation, while the estimated RNA velocity in turn refines the direction of pseudotime. By benchmarking InterVelo against existing methods on both simulated and real datasets, we demonstrate its superior performance in recovering pseudotime and RNA velocity. InterVelo yields more precise velocity estimations in terms of both direction and magnitude, with outstanding robustness across diverse scenarios. Furthermore, it successfully identifies driver genes and enables reliable gene activity enrichment analysis. The flexible architecture of InterVelo also allows for the integration of multi-omic data, enhancing its applicability to complex biological systems.

**Availability and implementation:**

InterVelo is implemented using Python, and the code is available on GitHub https://github.com/yurouwang-rosie/InterVelo and has been archived with a DOI https://doi.org/10.5281/zenodo.16158798 for reproducibility.

## 1 Introduction

Single-cell RNA sequencing facilitates the analysis of gene expression at single-cell resolution. However, it provides data solely as a static snapshot (Pan and Zhang 2024). Although methods such as metabolic labeling have been developed to learn cell state transitions from single-cell sequencing data, their application is confined to a limited number of biological systems (Baron and van Oudenaarden 2019, Erhard *et al.* 2019, Battich *et al.* 2020). To address this challenge, various computational approaches have been proposed to uncover the cellular trajectory, which can be broadly classified into two categories: pseudotime ordering-based and RNA velocity-based trajectory inference.

Pseudotime inference typically ranks cells based on cell similarity (Qiu *et al.* 2017, Street *et al.* 2018, Setty *et al.* 2019, Qu *et al.* 2025). Although these methods are flexible and extensible, they present several limitations: (1) Most require prior knowledge, such as the root of the cell population, which may not always be accessible. (2) As trajectory inference functions as a black box, it lacks biological interpretability. (3) Trajectory inference is limited in its ability to capture the dynamics of individual genes.

Compared to pseudotime inference, RNA velocity methods offer greater interpretability and can capture the changes of individual genes along the trajectory. RNA velocity model deciphers the expression changes of unspliced and spliced messenger RNA (mRNA) during transcription, splicing and degradation using two ordinary differential equations (ODEs), where RNA velocity refers to the first derivate of spliced mRNA with respect to time. The two most widely adopted methods for RNA velocity modeling are the steady-state and dynamical models, which employ the least square method and expectation-maximization (EM) algorithm, respectively, to solve the ODEs (La Manno *et al.* 2018, Bergen *et al.* 2020). However, they give little consideration to uncertainty and treat genes independently with gene-specific times. To address these issues, veloVI employs a variational inference (VI) model that accounts for uncertainty and introduces a latent state to enable information sharing across genes (Gayoso *et al.* 2024). For computational simplicity, the methods discussed above assume constant transcription rates, which may be overly restrictive (Bergen *et al.* 2021). However, chromatin accessibility, protein abundance and methylation modifications all play roles in gene regulation and influence transcription rates (Clark *et al.* 2016). DeepVelo and cellDancer address this limitation by learning cell-specific kinetics through cell displacements (Cui *et al.* 2024, Li *et al.* 2024). MultiVelo extends the RNA velocity model by incorporating chromatin accessibility into the analysis (Li *et al.* 2023). Nevertheless, most RNA velocity models first compute the time of each gene individually and then integrate these into a unified cellular timeline, making the process indirect and potentially biased. scTour addresses this limitation by introducing a framework capturing the dynamics of cellular latent space and assigning cellular time directly (Li 2023). While scTour infers a more intuitive cellular pseudotime and extends its applicability to multi-omic data, it cannot compute the velocity of individual genes, which is crucial for understanding gene-specific dynamics and supporting downstream analyses (Qiu *et al.* 2020, Lange *et al.* 2022). Furthermore, scTour may infer incorrect pseudotime directions in the absence of prior information.

To address the aforementioned challenges, we propose a novel method named InterVelo, which simultaneously learns cellular pseudotime and RNA velocity. InterVelo begins by learning an unsupervised pseudotime to guide RNA velocity prediction, while the predicted velocity, in turn, supervises the estimation of pseudotime. The global time guidance eliminates the need to estimate gene-specific time, thereby reducing noise during velocity estimation. Additionally, InterVelo employs Euler’s method to enable a variable transcription rate along development, ensuring a more accurate velocity estimation. The direction of velocity then allows for the correction of pseudotime direction without requiring prior information, such as starting cells or developmental direction. Moreover, InterVelo is expandable to integrate multi-omic information. Compared to Multivelo, InterVelo does not rely on ODE assumptions about other omics, making it more flexible and broadly applicable beyond single-cell assays for transposase-accessible chromatin with high-throughput sequencing (scATAC-seq) data. To demonstrate its effectiveness, we applied InterVelo to a range of simulated and real datasets, including neurogenesis, pancreatic endocrinogenesis, and brain cortical formation data. The results highlight the accuracy and robustness of InterVelo in velocity and pseudotime estimation.

## 2 Materials and methods

### 2.1 The InterVelo architecture

**Figure 1.**
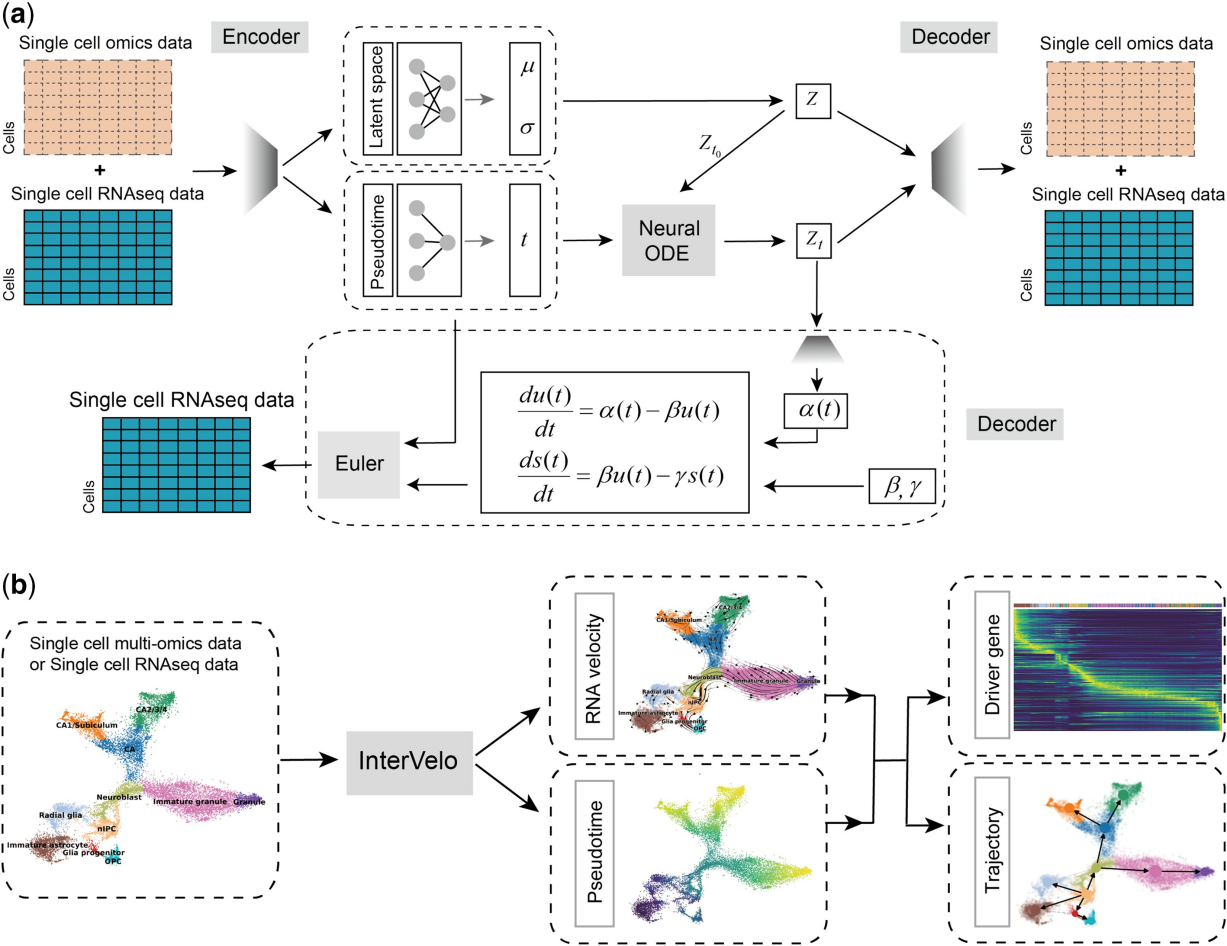
InterVelo architecture. (a) InterVelo framework: InterVelo takes single-cell RNA sequencing (scRNA-seq) data or multi-omics data as input. The model utilizes two encoders to generate both pseudotime t and posterior distribution parameters of the latent state Z. Then the initial state $Z_{t_{0}}$ and the time sequence${\;t}_{0},{\;t}_{1},\;\ldots ,{\;t}_{n}$ are encoded to produce another latent state $Z_{t}$. Both Z and $Z_{t}$ are fed into a decoder to reconstruct the input data. Additionally, $Z_{t}$ is utilized to decode $\alpha_{i,g}$, which alongside parameters $\beta_{g}$ and $\gamma_{g}$, predicts spliced and unspliced RNA velocities. Euler’s method is then used to reconstruct spliced and unspliced RNA expression based on the predicted velocities and pseudotime. (b) Application of InterVelo: InterVelo predicts both the pseudotime and RNA velocity of cells, which are utilized for downstream analyses, including trajectory reconstruction, identification of driver genes, and the exploration of primary cellular processes and gene regulatory mechanisms.

InterVelo is a deep learning-based generative model developed to predict RNA velocity under the guidance of cellular pseudotime. It comprises both unsupervised and supervised components (Fig. 1a). While the unsupervised component offers flexibility and captures cell state dynamics without kinetic assumptions, the supervised component introduces constraints by employing a model that approximates the biological basis of transcription dynamics.

The unsupervised component, adapted and modified from scTour (Li 2023), employs a variational autoencoder (VAE) (Kingma and Welling 2013) in conjunction with a neural ODE (Chen *et al.* 2018) to model the dynamics of the latent state, which is subsequently decoded to reconstruct the input data. The latent states Z and $Z_{t}$ are either sampled from the posterior distribution or generated from state dynamics (See Section 2.1.1 for details). Since the state dynamics are modeled without relying on kinetic assumptions, the unsupervised component adapts more effectively to real-world data and can be extended to capture state variability across multiple omic modalities.

The supervised component incorporates transcription dynamics assumptions to model the velocities of spliced and unspliced RNA. The processes of transcription, splicing and degradation are described as two ODEs:


$$\frac{dU_{i,g}}{dt}=\alpha_{i,g}-\beta_{g}U_{i,g},$$

$$\frac{dS_{i,g}}{dt}=\beta_{g}U_{i,g}-\gamma_{g}S_{i,g}.$$

To simplify the derivation of analytical solutions, most RNA velocity models fix the transcription rate $\alpha_{i,g}$ as a constant over developmental time. However, transcription rates are regulated by the transcriptional machinery and vary along the trajectory of cell differentiation (Weake and Workman 2010, Rada-Iglesias *et al.* 2011, Ostuni *et al.* 2013, Klemm *et al.* 2019). For instance, transcription factors (TFs) binding and chromatin compaction regulate transcription and lead to changes in $\alpha_{i,g}$ along development. Biases in $\alpha_{i,g}$ estimation also propagates to $\beta_{g}$ and $\gamma_{g}$. Therefore, we assume $\alpha_{i,g}$ varies with the cell state $Z_{t}$, as it encapsulates the regulation effect of cell machinery. To ensure flexibility and expandability, no specific functional form is assumed for how $Z_{t}$ influences $\alpha_{i,g}$. Instead of relying on analytical solutions, we approximate spliced and unspliced RNA expression using Euler’s method, guided by pseudotime (See Section 2.1.2 for details).

Compared to traditional RNA velocity models, InterVelo employs a global developmental time across genes for RNA estimation, which enhances velocity prediction. Traditional models assume that each gene fits in a specific phase portrait, making them prone to overfitting in the presence of technical noise. Moreover, these models initiate gene-specific time at the start of gene activation, which does not align with the overall developmental timeline. Consequently, deriving developmental time from gene-specific times introduces biases. InterVelo addresses this issue by directly predicting cellular time based on cell state dynamics, which improves interpretability, reduces biases, and enables the integration of multi-omic information. Compared to pseudotime inference methods, the pseudotime derived by InterVelo does not require prior information about developmental direction, as its direction is corrected by RNA velocity.

#### 2.1.1 Cellular pseudotime inference via VAE and neural ODE models

We denote the input matrix as $X\in R^{n\times m}$, where n is the number of cells and m is the number of features. If spliced and unspliced RNA sequencing data are input into InterVelo, then X=(S, U), where $S\in R^{n\times p}\;$denotes the spliced RNA matrix, $U\in R^{n\times p}\;$represents the unspliced RNA matrix. If additional omic data are available, the input matrix becomes X=(S, U, O), where $O\in R^{n\times h}\;$represents the additional omic matrix. Here, p is the gene number and h is the feature number in the other omics.

We use two encoders to reconstruct the input matrix. The first encoder, $f_{z}$, consists of two fully connected (FC) layers designed to encode the low dimensional latent variable $Z∼N(0,\;I_{d})$ that represents latent space of each cell. By default, d=20. We assume that the posterior q(Z|X) approximately follows a multivariate Gaussian distribution with a mean vector μ and a covariance matrix $\sigma^{2}I_{d}$, where $I_{d}$ is the identity matrix. Specifically,


Z=μ+σ⊙E,

where $\left(\mu ,\;\log\sigma^{2})=f_{z}(X\right)$ and $E∼N(0,I_{d}).$

The second encoder, $f_{t}$, consists of two FC layers and is designed to encode cellular pseudotime t and the pseudotime-dependent latent space $Z_{t}$. The first layer of $f_{t}$ is shared with $f_{z}$. The output of $f_{t}$ is scaled to the range (0,1) using a sigmoid function, representing the developmental time t. We rank the cells by sorting t and denote $Z_{t_{0}}$ as the initial latent state, which corresponds to $t_{n}$ at the first time point $t_{0}$. Assuming the latent space $Z_{t}$ evolves with t, we introduce another neural network, $f_{\mathrm{ode}}$, to capture the changing rate of $Z_{t}$. Using the rate from $f_{\mathrm{ode}}$, the pseudotime sequence $t_{0},t_{1},\ldots ,t_{n}$, and the initial state $Z_{t_{0}}$, we apply an ODE solver to compute $Z_{t_{1}},\;Z_{t_{2}},\;{\ldots ,\;Z}_{t_{n}}$:


$$t=f_{t}(X),$$

$$\frac{dZ_{t}}{dt}=f_{\mathrm{ode}}(Z_{t}),$$

$$Z_{t_{1}},Z_{t_{2}},{\ldots ,\;Z}_{t_{n}}=\mathrm{ODESolve}(Z_{t_{0}},\;f_{\mathrm{ode}},t_{0},t_{1},\;\ldots ,t_{n}).$$

The ODE solver is implemented using the *odeint* function from the torchdiffeq package (Chen *et al.* 2018). By default, the Euler method is used, which assumes the rate remains relatively stable within the small time interval between two adjacent pseudotime points:


$$Z_{t_{1}}=Z_{t_{0}}+f_{\mathrm{ode}}(Z_{t_{0}})\cdot (t_{1}-t_{0}),$$

$$Z_{t_{2}}=Z_{t_{1}}+f_{\mathrm{ode}}(Z_{t_{1}})\cdot (t_{2}-t_{1}),$$

…

$$Z_{t_{n}}=Z_{t_{n-1}}+f_{\mathrm{ode}}(Z_{t_{n-1}})\cdot (t_{n}-t_{n-1}).$$

After obtaining the latent space from the two encoders, we use the same decoder $f_{d}$ to reconstruct X from both Z and $Z_{t}$, respectively. $f_{d}$ is also a two-layer FC network.

The objective function of the unsupervised component is a modified lower bound consisting of three parts. The first part is the reconstruction error of Z and $Z_{t}$:

$L_{t1}=\omega \cdot \log p(X|Z)+(1-\omega )\cdot \log p(X|Z_{t})$

   $=-{\omega \cdot \|X-{\hat{X}}_{n}\|}_{2}^{2}-(1-\omega )\cdot {\left\|X-{\hat{X}}_{t}\right\|}_{2}^{2},$

where ω∈(0,1) is a weight.

The second part is the Kullback-Leibler (KL) divergence between the approximate posterior and prior:

$L_{t2}=-D_{KL}(q(Z|X)\|p(Z))$

$=-\int q(Z|X)\log\frac{q(Z|X)}{p(Z)}dZ.$

The third part is the mean squared error (MSE) between Z and $Z_{t}$:


$$L_{t3}=-{\left\|Z-Z_{t}\right\|}_{2}^{2}.$$

The objective function for cellular pseudotime estimation takes the form:


$$L_{t}=L_{t1}+L_{t2}+L_{t3}.$$

#### 2.1.2 RNA velocity estimation with a state-dependent transcription rate

Consider the following transcription dynamics:


$$\frac{dU_{i,g}}{dt}=\alpha_{i,g}-\beta_{g}U_{i,g},$$

$$\frac{dS_{i,g}}{dt}=\beta_{g}U_{i,g}-\gamma_{g}S_{i,g},$$

where $\alpha_{i,g}$ is the transcription rate of cell i and gene g, $\beta_{g}$ represents the splicing rate of gene g, and $\gamma_{g}$ denotes the degradation rate of gene g. The gene-specific rate equations depict the processes of transcription, splicing and degradation, which play a crucial role in modulating the expression levels of spliced and unspliced mRNA.

We define the velocity of unspliced RNA and spliced RNA as:


$$V_{i}^{u}\equiv {\left(\frac{dU_{i,1}}{dt},\;\frac{dU_{i,2}}{dt},\ldots ,\;\frac{dU_{i,p}}{dt}\right)}^{T},$$

$$V_{i}^{s}\equiv {\left(\frac{dS_{i,1}}{dt},\;\frac{dS_{i,2}}{dt},\ldots ,\;\frac{dS_{i,p}}{dt}\right)}^{T}.$$

Since spliced RNA plays a key role in gene regulation, its velocity is referred to as RNA velocity.

While $\beta_{g}$ and $\gamma_{g}$ are treated as constant parameters, the transcription rate α is allowed to vary dynamically in response to the cell state $Z_{t}$, with the expectation that α encapsulates underlying high-dimensional gene regulatory mechanisms. This modification aims to provide a more accurate representation of biological processes. Specifically, we employ a two-layer FC network $f_{\alpha }$ to model this relationship:


$$\alpha =f_{\alpha }(Z_{t}).$$

The modification to α introduces complexity to the ODE equations, rendering an analytical solution unattainable. To address this, we employ the Euler method to estimate the values of S and U:


$${\hat{X}}_{t_{1}}^{su}=X_{t_{0}}^{su}+\;\hat{V}(t_{0})\cdot (t_{1}-t_{0}),$$

$${\hat{X}}_{t_{2}}^{su}={\hat{X}}_{t_{1}}^{su}+\;\hat{V}(t_{1})\cdot (t_{2}-t_{1}),$$

…

$${\hat{X}}_{t_{l}}^{su}={\hat{X}}_{t_{l-1}}^{su}+\hat{V}(t_{l-1})\cdot (t_{l}-t_{l-1}),$$

where $X^{su}=(S,\;U)$, and $V(t)=(V^{s}(t),\;V^{u}(t))$. After sorting t, we choose $t_{0}$ at intervals of length l to mitigate the accumulated error introduced by the Euler method. By default, l=50.

The objective function for velocity estimation comprises the reconstruction error between $X^{su}$ and ${\hat{X}}_{t}^{su}$:


$$L_{v1}=\log p(X^{su}|Z_{t})=-{\left\|X^{su}-{\hat{X}}_{t}^{su}\right\|}_{2}^{2},$$

along with a penalty on the unspliced RNA velocity:


$$L_{v2}=-\sum_{i,g\in \Omega }{\left\|V_{i,g}^{u}\right\|}_{2}^{2},$$

where Ω denotes the samples whose gene expression exceeds the 95th percentile. This penalty is introduced to restrict the flexibility of α, as genes in these samples tend to exhibit stability. Specifically, the objective function has the form:


$$L_{v}=L_{v1}+L_{v2}.$$

#### 2.1.3 Objective function

Overall, the objective function of the model is:


$$L=L_{t}+\lambda_{v}L_{v},$$

where $\lambda_{v}$ is a weight hyperparameter.

### 2.2 Initialization and optimization

The initial values of $\beta_{g}$ and $\gamma_{g}$ are sampled from a log-normal distribution with a mean of 0 and a standard deviation of 0.1. All neural networks are initialized using PyTorch’s default setting. Across all experiments, the loss weights are set as ω=0.5, $\lambda_{v}=1$. InterVelo utilized mini-batch training. The batch size is 1024 by default. Since the ODE solver operates on mini-batches, the step size is indirectly influenced by the choice of batch size. Our analysis of batch size effects indicates that velocity estimation results show remarkable stability across different batch sizes (Supplementary Note, available as supplementary data at *Bioinformatics* online).

To optimize the loss function L, we use the Adam optimizer with a learning rate of 0.01, a weight decay of 0.01, and an epsilon (eps) value of 0.01, with AMSGrad enabled.

### 2.3 Direction correction

After training, InterVelo applies a direction correction using Pearson correlation loss to address the issue where solving the ODE may simultaneously reverse both velocity and pseudotime directions:


$${\hat{X}}_{t_{l}}^{su}={\hat{X}}_{t_{l-1}}^{su}+\hat{V}(t_{l-1})\cdot \Delta t={\hat{X}}_{t_{l-1}}^{su}+(-\hat{V}(t_{l-1}))\cdot \Delta (1-t).$$

The Pearson correlation loss determines the velocity direction by leveraging the relationship between spliced and unspliced RNA. Notably, since ${\hat{V}}_{i,g}^{s}=\beta_{g}U_{i,g}-\gamma_{g}S_{i,g}$, ${\hat{V}}_{i,g}^{s}$ should be positively correlated with $U_{i,g\;}$ and negatively correlated with $S_{i,g}$. To adjust the direction, we introduce another component:


$$L_{\mathrm{Pearson}}=\lambda_{u}c\mathrm{orr}({\hat{V}}_{i}^{s},\;U_{i})-\lambda_{s}c\mathrm{orr}({\hat{V}}_{i}^{s}{,\;S}_{i}),$$

where $\lambda_{u},\;\lambda_{s}\in (0,1)$ are regularization weights, and corr represents the Pearson correlation coefficient. If $L_{\mathrm{Pearson}}<0$, the pseudotime and velocity will be reversed, that is, t=1-t, V=-V.

### 2.4 Data processing and comparative benchmarking

Details on data processing and comparative benchmarking between InterVelo and other methods are provided in the Supplementary Notes (available as supplementary data at *Bioinformatics* online).

## 3 Results

### 3.1 InterVelo achieves precise prediction of velocity and pseudotime

We assessed the performance of InterVelo to infer velocity and pseudotime through a series of tests on both simulated and real datasets.

First, we evaluated InterVelo’s ability to infer kinetic parameters using two simulated datasets with either linear or cycle trajectories, where $\beta_{g}$ and $\gamma_{g}$ were held constant, while $\alpha_{i,g}$ parameters varied throughout development (Supplementary Note 1, available as supplementary data at *Bioinformatics* online). We assessed InterVelo’s accuracy in pseudotime and RNA velocity estimation by comparing it to established methods, including the EM dynamical model scVelo, the deep learning model DeepVelo, and the Bayesian deep generative model veloVI. Since veloVI and DeepVelo lack the ability to independently infer pseudotime, we incorporated pseudotime inference methods (Monocle3, Slingshot and scTour) for comparison. Pseudotime inference methods were supplied with true root cells or predefined trajectory directions. Additionally, as a supplementary analysis, we employed scVelo’s built-in pseudotime inference function based on velocity estimations, which enabled us to compare the pseudotime accuracy of InterVelo with other RNA velocity methods.

**Figure 2.**
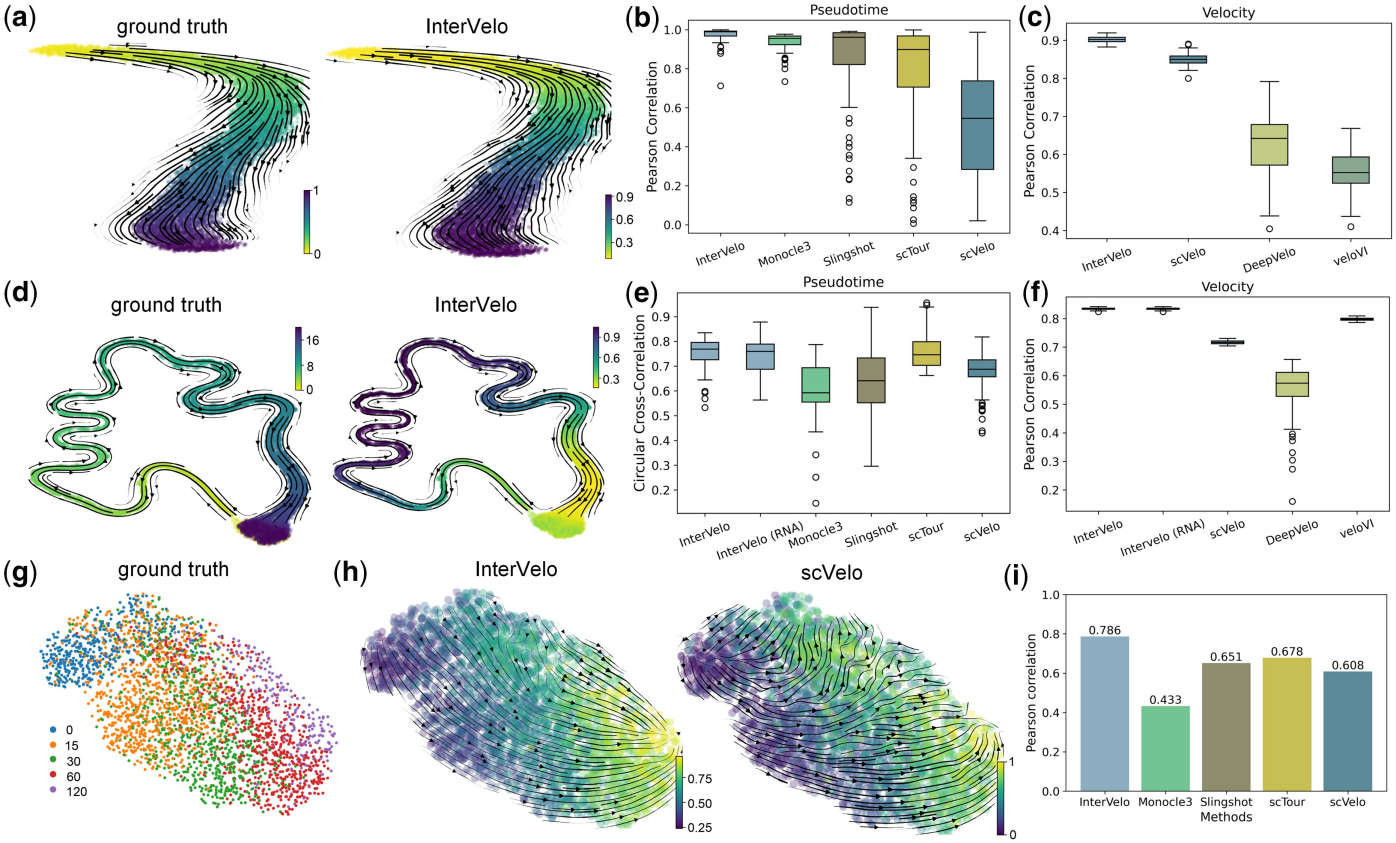
Benchmarking InterVelo on two simulated datasets and the metabolic labeled neuron data. (a, b, c) Comparison of InterVelo with other methods in single-omic dataset with a bell-shaped trajectory. (a) Streamlines of true velocity and velocity predicted by InterVelo are projected onto a Uniform Manifold Approximation and Projection (UMAP) embedding (Healy and McInnes 2024), with cells colored according to true time and pseudotime orders, respectively. (b, c) Boxplots of the Pearson correlation between predictions and ground truth for pseudotime and velocity, evaluated across 100 simulated datasets, comparing InterVelo to other methods. The velocity correlation is calculated as the average correlation of velocities across all genes within each sample. (d, e, f) Comparison of InterVelo with other methods on multi-omic state-switch data. (d) Streamlines of true velocity and velocity predicted by InterVelo are projected onto a UMAP embedding, with cells colored according to true time and pseudotime orders, respectively. (e, f) Boxplots of circular cross-correlation between predictions and ground truth for pseudotime and Pearson correlation for velocity, evaluated across 100 simulated datasets. InterVelo was tested under two conditions: (1) using only scRNA-seq data and (2) integrating scRNA-seq and scATAC-seq data. (g, h, i) Performance evaluation on metabolic labeled neuron data. (g) Ground-truth time of the metabolic labeled neuron data. (h) Velocity field predicted by InterVelo and scVelo, with cells colored by InterVelo’s pseudotime and scVelo’s latent time, respectively. (i) Pearson correlation between estimated pseudotime and true time.

In the single-omic dataset that has a bell-shaped trajectory, the velocity streamlines and pseudotime predicted by InterVelo and veloVI aligned well with the ground truth, whereas DeepVelo and scVelo exhibited incorrect directional predictions in parts of the trajectory (Fig. 2a, Supplementary Fig. 1a, available as supplementary data at *Bioinformatics* online). Among RNA velocity and pseudotime inference methods, InterVelo achieved the highest Pearson correlation in pseudotime estimation, suggesting that pseudotime provided reliable guidance for velocity estimation (Fig. 2b, Supplementary Fig. 1b, available as supplementary data at *Bioinformatics* online). Additionally, InterVelo ranked highest in velocity Pearson correlation among these RNA velocity models, demonstrating its effectiveness in estimating $\beta_{g}$ and $\gamma_{g}$ parameters when $\alpha_{i,g}$ varies along trajectory (Fig. 2c).

The second simulated dataset is a multi-omic state-switch dataset with a circular trajectory, generated using the EM dynamical model (Fig. 2d). For this circular dataset, we instead calculated the circular cross-correlation for pseudotime (Supplementary Note 1, available as supplementary data at *Bioinformatics* online). Although InterVelo is not designed for circular data, it achieved excellent performance in both pseudotime and velocity estimation across all methods tested (Fig. 2e and f). Integrating multi-omic data significantly enhanced InterVelo’s accuracy of pseudotime prediction, demonstrating its clear advantages over using scRNA-seq omics alone (Fig. 2e). Additionally, the spliced RNA velocity showed a high stability between single-omics and multi-omics, indicating that a rough pseudotime estimation is enough for InterVelo to estimate RNA velocity robustly (Fig. 2f).

To confirm the capability of InterVelo in velocity and pseudotime estimation, we analyzed metabolic labeled neuronal data from the scNT-seq study (Qiu *et al.* 2020), which provides ground-truth time information. In the study, primary mouse cortical neurons were labeled with 200 μM 4sU for 2 hours and then stimulated with potassium chloride (KCl)-mediated membrane depolarization for varying durations (0, 15, 30, 60, and 120 min) (Fig. 2g). InterVelo and DeepVelo accurately predicted cellular velocity directions, while veloVI produced reversed velocity flows and scVelo generated skewed predictions (Fig. 2h, Supplementary Fig. 2, available as supplementary data at *Bioinformatics* online). We further calculated the Pearson correlation between each method’s estimated pseudotime and the ground truth. InterVelo demonstrated superior performance compared to scVelo, Monocle3, Slingshot, and scTour (Fig. 2i, Supplementary Fig. 2, available as supplementary data at *Bioinformatics* online).

### 3.2 InterVelo unravels cellular fates and genetic dynamics

**Figure 3.**
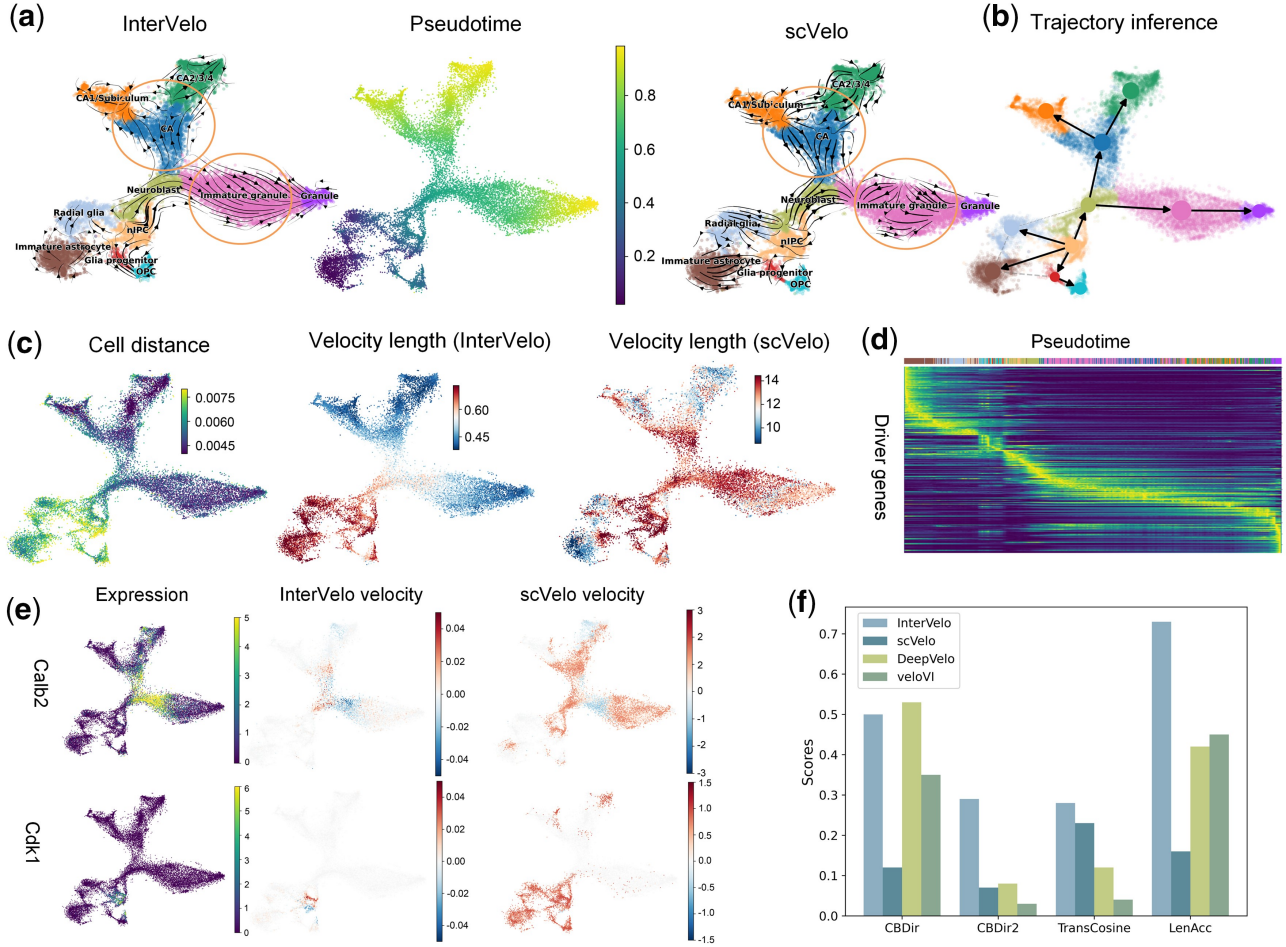
InterVelo unravels cellular fates and genetic dynamics in mouse dentate gyrus development (P0+P5). (a) Comparison of InterVelo with the dynamical model scVelo. The velocity streamlines and pseudotime derived from InterVelo, along with the velocity streamlines from scVelo, are projected onto t-Distributed Stochastic Neighbor Embedding (t-SNE) (van der Maaten and Hinton 2008) plots. (b) The trajectory inferred by PAGA using InterVelo’s velocity. (c) t-SNE plots of cell distance, velocity length estimated by InterVelo and velocity length estimated by scVelo, respectively. (d) Heatmap showing the top 30 driver genes for each cell type. Driver gene expression is plotted along pseudotime. (e) Gene expression and velocities predicted by InterVelo and scVelo for Calb2 and Cdk1 genes are projected onto t-SNE plots. (f) Histograms of CBDir, CBDir2, TransCosine and LenAcc matrics for InterVelo, DeepVelo, veloVI and scVelo.

Next, we evaluated InterVelo’s capacity to infer cell trajectory and gene kinetics in a multi-branch mouse hippocampal dentate gyrus development dataset from postnatal day 0 (P0) and P5 (Hochgerner *et al.* 2018). This dataset includes five branching lineages, from neurogenic intermediate progenitor cells (nIPC) to (1) oligodendrocyte precursors (OPCs), (2) astrocytes, (3) granule neurons, (4) pyramidal neurons in Cornu Ammonis region 1 (CA1) and subiculum, and (5) pyramidal neurons in CA2, CA3 and CA4 (Malatesta *et al.* 2003, La Manno *et al.* 2018). We applied both InterVelo and scVelo to predict RNA velocity in this context. With the aid of cellular pseudotime, InterVelo successfully delineated a precise velocity direction, while scVelo failed to capture the right developmental orders in CA1, subiculum, CA2, CA3, CA4 and granule lineages (Fig. 3a, Supplementary Fig. 3a, available as supplementary data at *Bioinformatics* online). In pseudotime prediction, InterVelo also demonstrates superior performance compared to pseudotime inference methods Monocle3, Slingshot and scTour (Supplementary Fig. 4, available as supplementary data at *Bioinformatics* online). The velocity computed by InterVelo is proved sufficient for trajectory inference, accurately reflecting the true developmental trajectory (Fig. 3b). Additionally, InterVelo’s velocity length showed a stronger coincidence with cell displacement, highlighting its superior precision in velocity prediction (Fig. 3c).

To assess the efficiency of InterVelo in characterizing genetic profiles, we identified the driver genes of each cluster using the velocity by InterVelo (Supplementary Note 2, available as supplementary data at *Bioinformatics* online, Fig. 3d). InterVelo successfully identified function-related genes and accurately predicted their expression trends. For example, Ntm (a neurite outgrowth-promoting cell adhesion molecule (Parikh *et al.* 2020, Liau *et al.* 2023)) showed high expression in CA1, subiculum, immature astrocytes, and OPCs. Velocity predictions of InterVelo aligned with Ntm’s expression variability, whereas scVelo produced inconsistent velocities (e.g., artificially high signals in Ntm-low regions and underestimation in Ntm-high regions). Nfib (critical for dentate gyrus formation (Barry *et al.* 2008)) velocities predicted by InterVelo matched its nIPC-specific expression, while scVelo overestimated velocities broadly (Supplementary Fig. 3c and d, available as supplementary data at *Bioinformatics* online).

**Figure 4.**
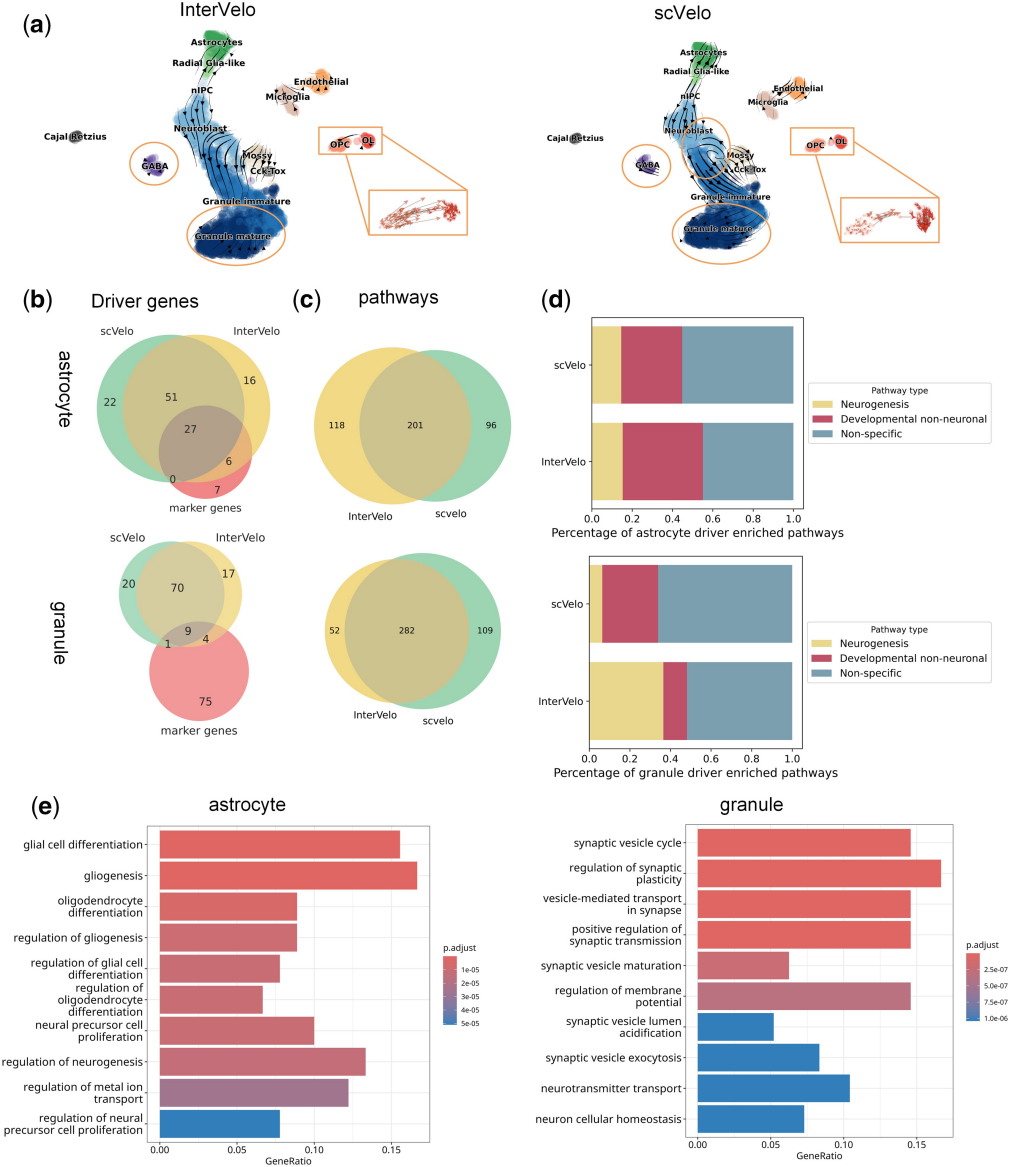
InterVelo infers driver genes and key processes in the cerebellum. (a) The streamlines of velocity derived from InterVelo and scVelo are visualized on UMAP coordinates. The velocity from OPCs to OLs is amplified for clarity. (b) Overlap analysis between putative driver genes and marker genes for two lineages. Marker genes were filtered to include only those within the top 2,000 variable genes. (c) Overlap of enriched pathways in two lineages derived from InterVelo and scVelo. (d) Ratio of pathway types among inconsistent pathways identified by InterVelo and scVelo. (e) Top 10 enriched BP pathways for astrocyte and granule lineages identified by InterVelo, ranked by adjusted p-value.

We also validated the velocity field consistency of InterVelo using two marker genes, Calb2 (encoding calretinin, an early neurogenesis marker (Sellers *et al.* 2014)) and Cdk1 (a cell cycle regulator enriched in nIPCs (Massacci *et al.* 2023)), whose expression patterns have been experimentally validated by multiplexed RNA staining of the dentate gyrus (Hochgerner *et al.* 2018). InterVelo’s predicted velocities for these markers aligned with their expected transcriptional dynamics (Fig. 3e), whereas scVelo produced spurious velocities in clusters lacking marker gene activation. The compatibility between velocity and expression variability indicates that InterVelo is reliable in capturing gene expression patterns and identifying driver genes. Further discussion of its ability to identify driver genes is provided in Fig. 4.

We used four metrics to furthermore assess the performance of InterVelo, including cross-boundary direction correctness (CBDir), CBDir2, transition cosine similarities (TransCosine) and length accuracy (LenAcc). The CBDir, CBDir2 and TransCosine metrics evaluate the direction of velocity from different perspectives, while LenAcc evaluates the length of velocity (Supplementary Note 3, available as supplementary data at *Bioinformatics* online) (Zheng *et al.* 2023). InterVelo consistently outperformed or at least matched scVelo, DeepVelo and veloVI, highlighting its superior ability to accurately characterize cell trajectories and genetic profiles in the dentate gyrus dataset (Fig. 3f). These evaluations were also conducted in other datasets discussed in the article, consistently demonstrating the superior performance of InterVelo in velocity estimation (Table 1).

**Table 1.** The four metrics to measure the accuracy of velocity direction and length in the four datasets discussed in Figs 3–6, respectively.

Datasets	Methods	CBDir	CBDir2	Trans-Cosine	LenAcc	Mean
Metabolic labeled neuronal data	InterVelo	0.35	0.32	0.37	0.63	0.42
	DeepVelo	0.33	0.11	0.15	0.18	0.19
	veloVI	0.01	0.05	0.06	0.44	0.14
	scVelo	0.21	0.01	0.12	0.12	0.12
Dentate gyrus P0+P5 (Fig. 3)	InterVelo	0.50	0.29	0.28	0.73	0.45
	DeepVelo	0.53	0.08	0.12	0.42	0.34
	veloVI	0.35	0.03	0.04	0.45	0.28
	scVelo	0.12	0.07	0.23	0.16	0.12
Dentate gyrus P12+P35 (Fig. 4)	InterVelo	0.43	0.25	0.28	0.71	0.46
	DeepVelo	0.32	0.15	0.18	0.31	0.26
	veloVI	0.37	0.06	0.06	0.27	0.23
	scVelo	−0.10	0.03	0.11	0.29	0.07
Pancreas	InterVelo	0.38	0.22	0.23	0.63	0.41
	DeepVelo	0.35	0.05	0.10	0.20	0.20
	veloVI	0.33	0.06	0.06	0.30	0.23
	scVelo	0.29	0.10	0.14	0.14	0.18
Cortex	InterVelo	0.24	0.35	0.36	0.52	0.37
	DeepVelo	0.24	0.01	0.06	−0.34	−0.03
	veloVI	0.10	0.08	0.08	−0.32	−0.05
	scVelo	0.20	0.06	0.11	0.54	0.27

### 3.3 InterVelo infers driver genes and key processes in cerebellum

To further validate the reliability of InterVelo for identifying driver genes, we applied InterVelo to another scRNA-seq dataset of postnatal neurogenesis in the dentate gyrus, sampled at two experimental time points: P12 and P35 (Hochgerner *et al.* 2018). The neurogenesis dataset mainly captures the progression from nIPCs to granule cells (Hochgerner *et al.* 2018). While scVelo produced a reversed flow from neuroblasts to immature granules, InterVelo accurately inferred the developmental direction (Fig. 4a). Notably, both models identified a developmental lineage from radial glia to astrocytes, a finding that was not confirmed in the original research (Bergen *et al.* 2020). In addition to the main branches, the dataset includes scattered cell groups that stay at terminal states, including Cajal–Retzius (CR) cells, GABA cells and oligodendrocytes (OLs) (Bergen *et al.* 2020). Their states are more closely aligned with the velocities inferred by InterVelo. InterVelo also identified the terminal state of mature granules. Furthermore, InterVelo demonstrated greater consistency with the developmental trajectory from OPCs to OLs (Beyer *et al.* 2018).

To infer the driver genes, we utilized CellRank2 (Reuter *et al.* 2019, Weiler *et al.* 2024), which integrates the velocity and pseudotime computed by InterVelo or scVelo, with a focus on the driver genes associated with two lineages: the granule lineage and the astrocyte lineage. Genes were ranked based on lineage fate correlations, and the top 100 genes were selected as lineage driver genes (Supplementary Note 2, available as supplementary data at *Bioinformatics* online). To validate the reliability of identified driver genes, we compared them to a reference marker gene set derived from the dataset’s original research (Hochgerner *et al.* 2018). The driver genes identified by InterVelo showed a better alignment with the reference marker gene set for both the astrocyte and granule lineages (Fig. 4b). We further examined the driver genes uniquely identified by InterVelo that did not overlap with those identified by scVelo. Most of these genes displayed an organized distribution along the developmental trajectories (Supplementary Fig. 5a and b, available as supplementary data at *Bioinformatics* online). To assess the gene rankings, we checked the ranks of each marker gene among the top 2,000 variable genes. Since the dataset primarily focuses on the granule lineage, identifying its driver genes was relatively straightforward. No distinct difference was observed in marker gene rankings between InterVelo and scVelo for the granule lineage. However, for the astrocyte lineage, InterVelo assigned higher rankings to marker genes compared to scVelo, demonstrating its superior ability to capture signals in this context (Supplementary Fig. 5c, available as supplementary data at *Bioinformatics* online).

We further evaluated the reliability of InterVelo through Gene Ontology Biological Process (GO BP) pathway enrichment analysis for astrocyte and granule lineages, using the top 100 driver genes identified by InterVelo and scVelo (Supplementary Note 4, available as supplementary data at *Bioinformatics* online). Most of the pathways enriched by InterVelo demonstrated high consistency with those identified by scVelo (Fig. 4c). To explore the pathway enrichment discrepancies between the two methods, we extracted the inconsistent pathways and analyzed their relevance to neurogenesis and development. InterVelo exhibited superior performance in capturing pathways closely associated with these processes (Fig. 4d). More precisely, the pathways enriched for astrocyte and granule lineages by InterVelo were strongly correlated with gliogenesis and synaptic plasticity, respectively, aligning well with the expected biological processes for these lineages (Fig. 4e) (Frisén 2016, Accogli *et al.* 2020). These findings suggest that InterVelo provides more reliable results for identifying driver genes and performing pathway enrichment analyses.

### 3.4 InterVelo is robust against missing cell types, low sequencing depth and read loss

**Figure 5.**
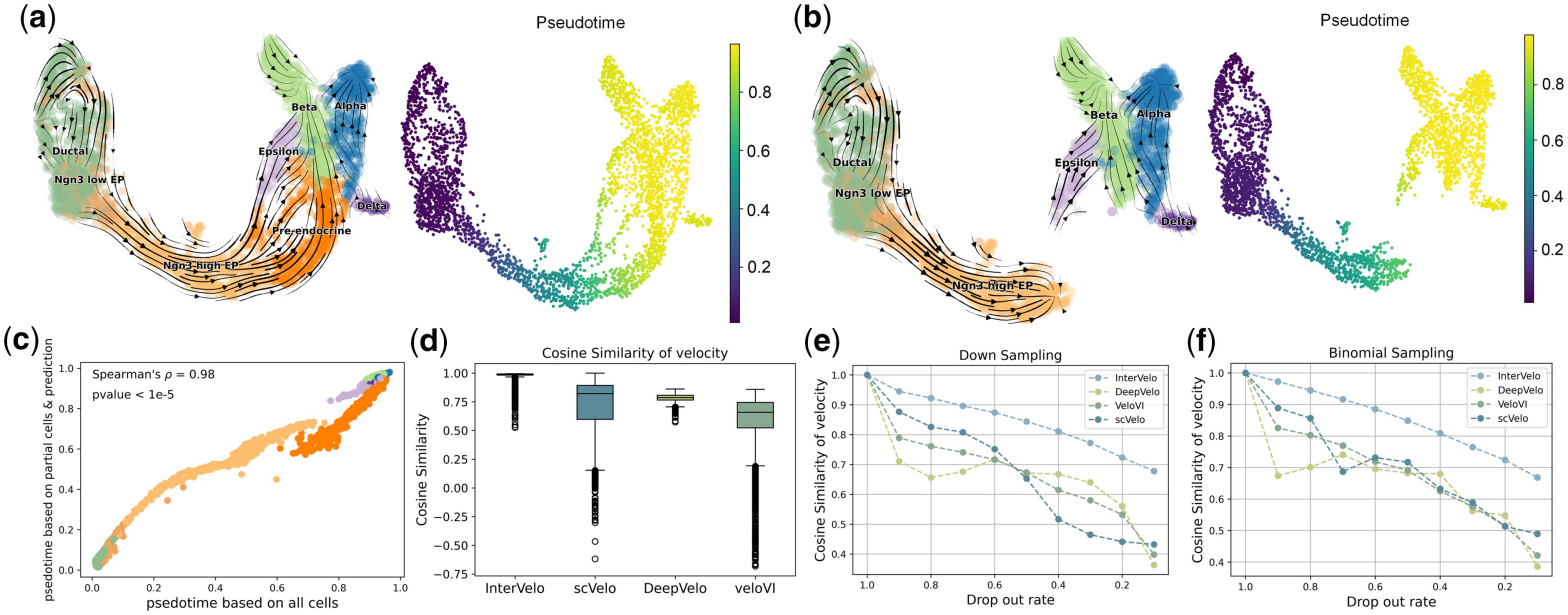
InterVelo demonstrates excellent stability against multiple disturbances in pancreas endocrinogenesis. (a) Velocity and pseudotime derived from InterVelo using the complete dataset are projected onto the UMAP plot. (b) Velocity and pseudotime derived from InterVelo using the partial dataset are projected to the UMAP plot. (c) Scatter plot comparing pseudotime estimates from the complete dataset and the partial dataset. (d) Boxplot showing cosine similarity between velocity derived from the complete dataset and partial dataset across different methods. (e, f) Line charts depicting the mean of cosine similarity between velocity derived from the complete dataset and down-sampling or binomial sampling datasets.

To test if InterVelo is effective in denoising for a more stable velocity estimation, we conducted a series of experiments using the mouse pancreas endocrine development transcriptome data sampled from embryonic day 15.5 (E15.5) (Bastidas-Ponce *et al.* 2019). The pancreas dataset transitions from ductal cells to alpha, beta, delta and epsilon cells (Byrnes *et al.* 2018). InterVelo accurately predicted the cycling developmental structure (Bergen *et al.* 2020) in ductal progenitor cells and the smooth flow of cell differentiation (Fig. 5a).

To assess the stability of the velocity estimation, we disturbed the dataset in several ways. Since the presence of technical noise, data missing or insufficient sequencing depth may result in certain cell populations incompletely sampled (Wolf *et al.* 2019), we simulated a disconnected trajectory by removing the pre-endocrine cells from the pancreas dataset. InterVelo’s model trained on the partial dataset was used to predict pseudotime of the entire dataset. The pseudotime estimation was highly correlated with the pseudotime predicted from the complete dataset, with a Spearman’s correlation of 0.98 (Fig. 5b and c). This indicates that, despite the absence of intermediate data, InterVelo still provided a stable estimation of pseudotime, offering reliable coordinates for velocity estimation. Additionally, we computed velocity based on the model trained on the partial dataset. Compared to other methods, InterVelo demonstrated greater stability in velocity estimation for the partial dataset, as the velocity derived from the partial dataset closely resembled that from the complete dataset (Fig. 5d, Supplementary Fig. 6, available as supplementary data at *Bioinformatics* online).

Next, to further ascertain the robustness of velocity estimation under different measurement conditions, we conducted two simulations using the pancreas dataset: (1) down-sampling to simulate low sequencing depth, and (2) binomial sampling to mimic read loss in low-quality data (Supplementary Note 5, available as supplementary data at *Bioinformatics* online). InterVelo showed consistent superiority over other methods in velocity stability across different dropout rates (Fig. 5e and f). This suggests that, with the guidance of global time, InterVelo offered a more robust and reliable RNA velocity estimation across various sequencing conditions.

### 3.5 InterVelo exhibits the capability to integrate multi-omic information

InterVelo is a flexible framework capable of incorporating multi-omic information. While its advantages in incorporating multi-omic data have been validated on a simulated dataset, we further evaluated its performance on a developing human cortex multi-omic dataset that simultaneously measures gene expression and chromatin accessibility within the same cell (Trevino *et al.* 2021). The cortex originates from a group of cycling cells that differentiate into basal radial glia (RG). RG cells can subsequently develop into intermediate progenitor cells (IPCs) or differentiate into astrocytes and OPCs. nIPCs, a type of transit-amplifying cell, are responsible for producing excitatory neurons (ExNs). During development, nIPCs and ExNs migrate from the proliferative zone to the cortical plate, forming the layered structure of the cortex (Molnár *et al.* 2019), with these cells referred to as excitatory maturing cells (ExMs). Depending on the cortical layer where they ultimately settle, the neurons are classified as excitatory upper-layer neurons (ExUp) or excitatory deep-layer neurons (ExDp). Notably, the formation of ExDp occurs later than that of ExUp (Trevino *et al.* 2021).

**Figure 6.**
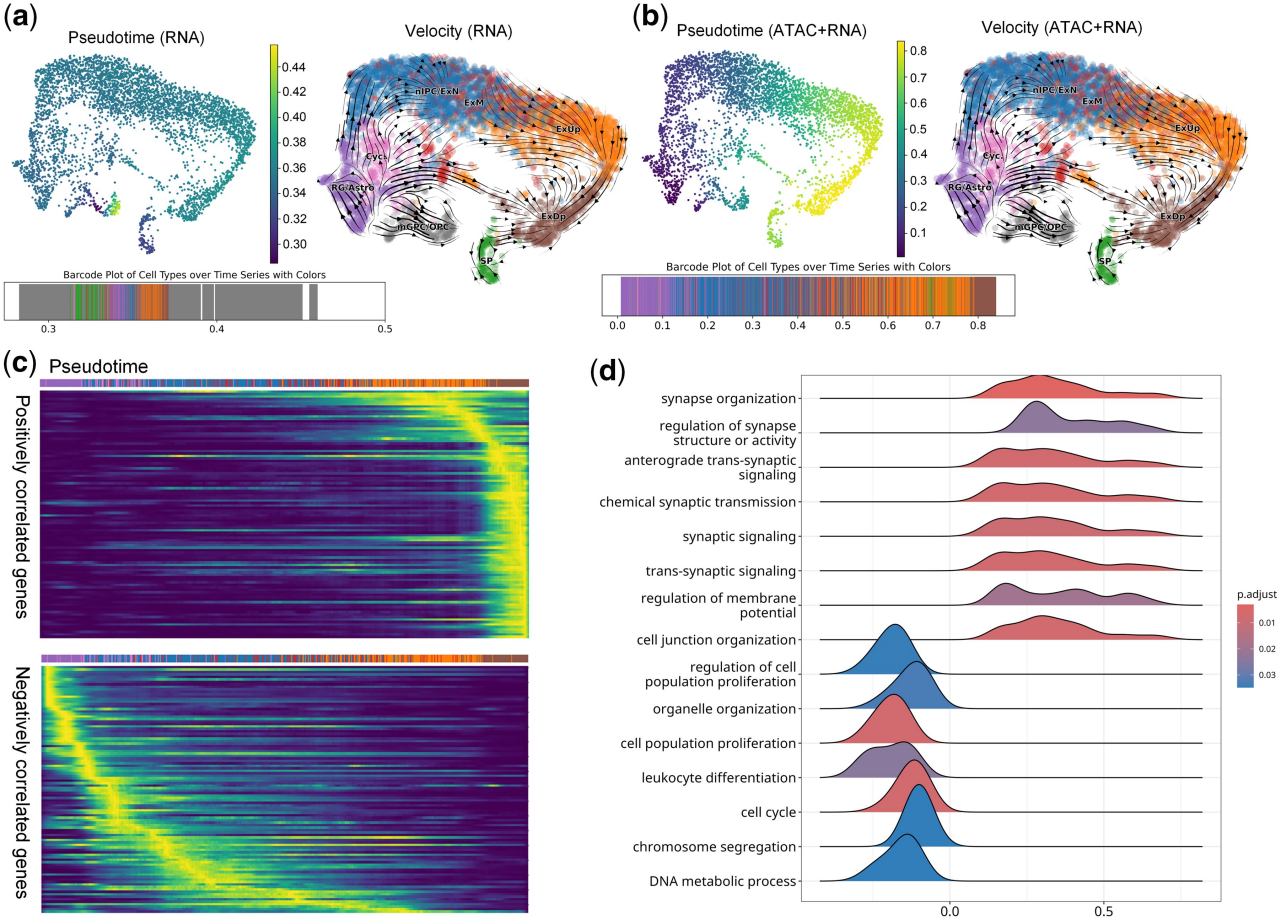
InterVelo exhibits the capability to integrate multi-omic information. (a, b) Pseudotime and velocity streamflows are projected onto UMAP plots generated by InterVelo using (a) scRNA-seq data alone and (b) scATAC-seq+scRNA-seq data. Colors in barcode plots correspond to cell types. mGPC/OPC, multipotent glial progenitor cells/oligodendrocyte progenitor cells; Cyc., cycling progenitors; RG/Astro, radial glia/astrocytes; SP, subplate. (c) Heatmaps showing the expression of the top 100 positively and negatively correlated genes along the neurogenesis lineage, identified by InterVelo. The heatmaps are restricted to cells of the neurogenesis lineage. (d) Ridge plot of GSEA enrichment results, with pathways ordered by Normalized Enrichment Score (NES).

We used scRNA-seq data and multi-omic data to train InterVelo, respectively. Since the trajectory of subplate and mGPC/OPC clusters diverges from the main lineage, ordering these clusters led to discrepancies in pseudotime estimation based solely on scRNA-seq data. Although InterVelo could approximate cell ordering along the main branch, the majority of the time span was dominated by the subplate and mGPC/OPC clusters (Fig. 6a). However, incorporating scATAC-seq data improved the accuracy of pseudotime prediction (Fig. 6b). Despite minor discrepancies in pseudotime estimation for single-omics data, the predicted velocity remained robust and accurate (Fig. 6a and b), indicating that moderate error in pseudotime inference has minimal impact on velocity estimation. Moreover, despite not explicitly accounting for epigenomic dynamics, InterVelo outperformed MultiVelo, which exhibited a reflux velocity during the transition from ExM to ExUp (Supplementary Fig. 7a, available as supplementary data at *Bioinformatics* online).

We next evaluated whether InterVelo can detect signals of gene activity in the cortex dataset. Using the CellRank2 package, we identified the top 100 positively and negatively neurogenesis correlated genes among the 2,000 variable genes. These genes were shown to be up-regulated and down-regulated along the trajectory, respectively, indicating that InterVelo effectively captures gene dynamics (Fig. 6c, Supplementary Fig. 7b, available as supplementary data at *Bioinformatics* online). Based on lineage correlations computed by CellRank2, we performed GSEA enrichment analysis on the 2,000 variable genes using GO BP databases. The up-regulated pathways were strongly associated with synaptic organization and signaling, predominantly expressed in ExUp and ExDp. In contrast, the down-regulated pathways were linked to cell cycle and proliferation, primarily expressed in cycling cells and nIPCs (Trevino *et al.* 2021) (Fig. 6d).

## 4 Discussion

InterVelo is a novel framework designed to estimate cellular pseudotime and RNA velocity simultaneously, enabling these two components to mutually enhance one another. While velocity provides critical information to refine the direction of unsupervised pseudotime, the global pseudotime helps reduce noise in velocity estimation. Traditional methods calculate time for each gene individually (Bergen *et al.* 2020, Gayoso *et al.* 2024), which introduces complexity and noise. In contrast, InterVelo employs an unsupervised approach to estimate pseudotime by assuming a state flow, which effectively captures overall dynamics, denoises and allows for seamless integration of information from additional omics layers. Considering the critical role of other omics in gene regulation, InterVelo is designed to integrate multi-omic data for velocity estimation. To maintain flexibility, no constraints are imposed on how other omics influence the transcription rate. However, the transcription rate, which is derived from the cell state, likely reflects underlying high-dimensional gene regulatory mechanisms. By comparing InterVelo to several existing methods using both simulated and real datasets, we validated its advantage. The reliable pseudotime enables velocity estimation with greater accuracy in both direction and magnitude, making it well-suited for trajectory inference, gene profiling, driver gene analysis, and pathway enrichment. Furthermore, the velocity estimation demonstrated exceptional robustness under various noise conditions, confirming the reliability of InterVelo.

Although the robustness and expandability of InterVelo have been validated, the model still has limitations. InterVelo assumes a continuous ODE-based trajectory, which may limit performance on purely cyclic or disconnected systems. Owing to the shared assumptions underlying ODE-based models, other RNA velocity methods are also vulnerable to substantial discontinuities, highlighting the need for flexible modeling approaches. However, the use of fixed β and γ parameters allows for robust velocity estimation even in cyclic regions—particularly when guided by locally progressive expression trends. For instance, InterVelo successfully captured the cyclic trajectory of ductal cells (Fig. 5a). Additionally, as demonstrated in Fig. 5, minor gaps or discontinuities in the trajectory do not significantly impact the performance, indicating the flexibility and robustness of InterVelo. Secondly, InterVelo’s pseudotime estimation depends on the initial pseudotime values. Though RNA velocity can help correct the pseudotime direction, a poor initial estimate that deviates substantially from the true trajectory may bias pseudotime inference. However, our experiments show that the velocity estimation remains robust across different initializations, largely due to the fixed parameters $\beta_{g}$ and $\gamma_{g}$. Consequently, we designed a criterion to ensure consistent alignment between estimated pseudotime and velocity field: using estimated RNA velocity rates, we estimate the velocity pseudotime with scVelo’s function *scv.tl.velocity_pseudotime* and assess its Pearson correlation with the estimated pseudotime. If the correlation is negative, we reverse the direction of the initial pseudotime values and retrain the model. Applying this criterion allows us to achieve reliable pseudotime estimates in the majority of cases.

In our framework, we allow transcription rates to vary along developmental time while fixing splicing and degradation rates across cells. This design reflects a trade-off between biological realism and model stability. Our decision to fix these rates is consistent with common practices in RNA velocity modeling (Gao *et al.* 2022, Lederer *et al.* 2024, Aivazidis *et al.* 2025) and is supported by biological evidence suggesting that transcription rates are more dynamically regulated during development than splicing and degradation (Weake and Workman 2010, Rada-Iglesias *et al.* 2011). Nevertheless, incorporating variable splicing and degradation rates could provide valuable developmental insights, making this an important yet challenging direction for future research. Additionally, while InterVelo has demonstrated the ability of multi-omic data integration, there remains potential to explore higher-order gene interactions in velocity regulation across multi-omics (Singh *et al.* 2024). Addressing these challenges will be the focus of future work.

## Supplementary Material

btaf500_Supplementary_Data

## Data Availability

The datasets utilized in this study are publicly available. The annotated data of metabolic labeled neuronal data can be obtained from the dynamo study (Qiu *et al.* 2022). The raw data are available in the National Center for Biotechnology Information’s Gene Expression Omnibus repository (GEO) under accession number GSE141851 (Qiu *et al.* 2020). The annotated data of two hippocampal dentate gyrus and pancreatic endocrinogenesis can be accessed from https://scvelo.org. The raw data of the two hippocampal dentate gyrus datasets are available under accession number GSE95753 (Hochgerner *et al.* 2018). The raw data of pancreatic endocrinogenesis is available under accession number GSE132188 (Bastidas-Ponce *et al.* 2019). The annotated multi-omic human cortex data is from the MultiVelo paper (Li *et al.* 2023), with the raw data available under accession number GSE162170 (Trevino *et al.* 2021).
