# Supplementary material for: InterVelo: a mutually enhancing model for estimating pseudotime and RNA velocity in multi-omic single-cell data
Source: Bioinformatics. 2025 Sep 10;41(10):btaf500. doi: 10.1093/bioinformatics/btaf500 (PMC12552101; doi:10.1093/bioinformatics/btaf500)
Supplement: btaf500_Supplementary_Data [file btaf500_supplementary_data.zip › S1 Supplementary Notes.pdf]

S1: Supplementary Notes and Supplementary Figures.

1. Benchmarking between InterVelo and other methods

We employed two simulated datasets to assess the performance of InterVelo in comparison with other methods.

We utilized the UniTVelo model [1] to simulate the single-omic data with a bell-shaped trajectory:

$$S_{i,g}(t_n) = h_g e^{-a_g(t_n-\tau_g)^2},$$
$$U_{i,g}(t_n) = \frac{S'_{i,g}(t_n) + \gamma_g S_{i,g}(t_n)}{\beta_g}.$$

We simulated 100 datasets, each consisting of 10,000 cells across 1,000 genes. Gene-specific parameters $\{a_g, h_g, \beta_g, \gamma_g\}$ were sampled from a multivariate log-normal distribution with a mean of $(-2, 2, 0, 0)^{\mathrm{T}}$ and a covariance matrix with diagonal values of 0.5, 1, 0.3, and 0.3. The time intervals Δt were generated as $-0.1 \times \log U(0,1)$, where $U(0,1)$ denotes the uniform distribution on $(0,1)$. The times t were scaled to the range $(0,1)$. The gene-specific parameters τ_g were sampled from $U(-1,2)$. After generating the data, noise was added from a normal distribution with a mean of 0 and a standard deviation of 0.08 times the maximum value. Any negative values were set to 0.

To simulate multi-omic state-switch data, we used the model proposed in the MultiVelo paper [2]. Taking genetic accessibility $C_{i,g}$ into account, MultiVelo models transcription dynamics using the following ODEs:

$$\frac{dC_{i,g}}{dt} = K^c \alpha_g^c - \alpha_g^c C_{i,g},$$
$$\frac{dU_{i,g}}{dt} = \alpha_g^{(K)} C_{i,g} - \beta_g U_{i,g},$$
$$\frac{dS_{i,g}}{dt} = \beta_g U_{i,g} - \gamma_g S_{i,g},$$

where

$$K^c = \begin{cases} 1, \ if\ chromatin\ is\ opening, \\ 0, \ if\ chromatin\ is\ closing, \end{cases}$$

and

$$\alpha_g^{(K)} = \begin{cases} \alpha_g, & if\ induction\ (K = 1), \\ 0, & if\ repression\ (K = 0). \end{cases}$$

Based on the analytical expressions of $S_{i,g}$, $U_{i,g}$, and $C_{i,g}$, we simulated 100 datasets, each consisting of 10,000 cells across 1,000 genes. Gene-specific parameters $\{\alpha_g^c, \alpha_g, \beta_g, \gamma_g\}$ were sampled from a multivariate log-normal distribution with a mean of $(-2,\ 2,\ 0,\ 0)^{\mathrm{T}}$ and a covariance matrix with diagonal values of 0.5, 1, 0.3, and 0.3. The time intervals Δt were sampled from $-0.1 \times \log U(0,1)$. The times t were scaled to the range $(0, 20)$. The transition times for state K were sampled from $U(2, 10)$, while the transition times for chromatin state K^c were set to half the induction or repression time. After generating the data, we introduced noise from a normal distribution with a mean of 0 and a variation of 0.08 times the maximum value, adjusting any negative values to 0.

To evaluate the estimation accuracy of pseudotime and velocity, we calculated the Pearson correlation coefficient between the predictions and the ground truth. For velocity, Pearson correlations were computed for each gene, and the mean was calculated for each sample. For the circular multi-omic state-switch data, we used the circular cross-correlation to evaluate the pseudotime accuracy. The true time was scaled to the range $(0, 1)$ and shifted in increments of 0.1, wrapping around at 1. Pearson correlations were computed at each 0.1 increment, and the highest correlation value was selected as the final result.

2. Driver gene identification

In the hippocampal dentate gyrus dataset at P0 and P5, driver genes were identified using a Welch t-test with overestimated variance to assess velocity expression conservatively. This approach, conducted using the scVelo function *scvelo.tl.rank_velocity_genes*, aimed to detect genes differentially regulated in a specific cluster compared to all other clusters.

For the dentate gyrus dataset at P12 and P35, driver genes were identified following the standard procedure outlined by CellRank2 [3, 4]. First, three transition matrices

were computed using the functions *VelocityKernel*, *PseudotimeKernel* and *ConnectivityKernel,* respectively. These matrices were combined in a 0.6: 0.2: 0.2 ratio, emphasizing the contribution of velocity to driver gene inference. The combined matrix was then used to identify terminal states and compute the fate probability for each lineage. Genes were ranked based on their correlation with lineage development, and the top 100 positively correlated genes were selected as driver genes.

In the cortex multi-omic dataset, the processes used to compute lineage correlations for each gene kept the same as described above.

3. Evaluation Metrics

In the real dataset, four metrics were used to assess the accuracy of velocity estimation: CBDir, CBDir2, TransCosine, and LenAcc.

CBDir, CBDir2 and TransCosine, proposed in the VeloAE paper [5], measure how well the estimated velocity direction aligns with the ground truth of developmental direction from one cell type, A, to another, B. Under the assumption that a cell's nearest neighbors are those from the preceding or subsequent time points, the displacements from a type A cell to its neighboring type B cells are expected to align with the velocity direction. CBDir evaluates the velocity direction utilizing low-dimensional cell embedding displacements. For each boundary cell $c \in C_A$, it is defined as:

$$CBDir(c) = \frac{1}{|\{c' \in C_B \cap N(c)\}|} \sum_{c' \in C_B \cap N(c)} \frac{v_{e,c}(x_{e,c'} - x_{e,c})}{|v_{e,c}||x_{e,c'} - x_{e,c}|},$$

where C_A represents the set of type A cells, $N(c)$ denotes the k-nearest neighbors of cell c, $v_{e,c}$ is the embedding vector of velocity in cell c, $x_{e,c'}$ and $x_{e,c}$ represent the embedding vectors of spliced RNA expression in cell c' and c, respectively, obtained using the scVelo function *scvelo.pl.velocity_embedding_stream*.

CBDir2 assesses the direction at the gene level, replacing the UMAP vectors in CBDir with high dimensional gene expression x_c and velocity v_c:

$$CBDir2(c) = \frac{1}{|\{c' \in C_B \cap N(c)\}|} \sum_{c' \in C_B \cap N(c)} \frac{v_c(x_{c'} - x_c)}{|v_c||x_{c'} - x_c|}.$$

The TransCosine metric evaluates velocity direction in terms of velocity graph $\pi_{c,c'}$. $\pi_{c,c'}$ was proposed in scVelo paper and obtained using the scVelo function *scvelo.tl.velocity_graph* [6]. For each boundary cell $c \in C_A$, it is defined as:

$$TransCosine(c) = \frac{1}{|\{c' \in C_B \cap N(c)\}|} \sum_{c' \in C_B \cap N(c)} \pi_{c,c'}.$$

Following the DeepVelo paper [7], the average CBDir, CBDir2 and TransCosine for a dataset were calculated over all boundary cells rather than averaging metrics per cell-type pair. This mitigates the influence of cell number imbalance across cell-type pairs.

Although few methods focus on the velocity length, it is an important aspect for capturing development dynamics. Assuming uniform cell sampling and no significant biases in the number of cells from different time points, the cell distance is proportional to the velocity length. To compare the estimated velocity length with the ground truth, LenAcc is defined as:

$$LenAcc = \frac{\sum_{c=1}^{n}(d_c - \bar{d})(l_c - \bar{l})}{\sqrt{\sum_{c=1}^{n}(d_c - \bar{d})^2} \cdot \sqrt{\sum_{c=1}^{n}(l_c - \bar{l})^2}},$$

where d_c represents the mean cell distance between cell c and its neighboring cells, and l_c denotes the velocity length of cell c. The cell distance is calculated as the Euclidean distance to the k-nearest neighbors, obtained using the Scanpy function *scanpy.pp.neighbors* [8]. The velocity length of cell c is computed using the scVelo function *scvelo.tl.velocity_confidence*.

4. Pathway enrichment analysis

We performed the GO BP pathway enrichment analysis using the R package clusterProfiler (version 4.12.6) based on the top 100 driver genes [9, 10]. Pathways with a multiple-testing adjusted p-value below 0.05 were considered statistically significant. The enriched pathways were manually classified into three categories: "Neurogenesis," "Developmental non-neuronal" and "Non-specific." The "Neurogenesis" category includes entries related to neurogenesis, neuronal migration, and axon guidance [11], while entries related to organelle organization, cell development, differentiation, and

migration were classified under "Developmental non-neuronal." Pathways that were not directly related to development and did not fit the first two categories were grouped as "Non-specific."

The GSEA enrichment was conducted using clusterProfiler, ranking 2,000 variable genes based on lineage correlations computed by CellRank2.

5. Stability test against multiple disturbances

In the pancreatic endocrinogenesis data, we evaluated the stability of InterVelo under various disturbance scenarios. Velocity and pseudotime predictions using the full dataset served as the control group. First, we removed the pre-endocrine cells from the dataset and trained InterVelo on the modified data. The trained model was then used to predict pseudotime for the full dataset. To assess the stability of velocity estimation, cosine similarity was calculated between the control group and the modified dataset. Similarly, Spearman's correlation was used to evaluate the stability of pseudotime predictions.

Second, we simulated sampling deviations using two distinct downsampling methods. The first method employed the Scanpy function *scanpy.pp.downsample_counts* to simulate low-depth sequencing. The second method utilized the NumPy [12] function *np.random.binomial* to mimic read loss due to poor sequencing quality. Downsampling was performed in spliced and unspliced RNA with dropout ratios ranging from 0.1 to 0.9. InterVelo and other methods were then applied to predict velocity using the downsampled datasets, and the results were compared with the control group using cosine similarity.

6. Preprocessing the single cell multi-omic data for InterVelo and other methods

The input data of InterVelo included both spliced and unspliced RNA, with other omics data from the same cells being optional. The spliced and unspliced RNA data were preprocessed using standard data processing pipeline of scVelo [6]. Firstly, the spliced and unspliced RNA counts were normalized to the median of total molecules across all

cells. Genes with fewer than 20 counts (both spliced and unspliced) were filtered out, and the top 2,000 highly variable genes were subsequently selected. Then, the spliced and unspliced RNA data was smoothed based on the average of 30 nearest neighbors. The two steps are implemented using the scVelo functions *scvelo.pp.filter_and_normalize* and *scvelo.pp.moments*, respectively.

Smoothing is a crucial preprocessing step, as raw count data are often sparse and noisy. To assess the impact of smoothing, we conducted experiments on pancreas data using different numbers of neighbors (k = 20 and k = 30), as well as a setting without smoothing. Velocity estimates from non-smoothed data showed increased fluctuations and directional reversals. In contrast, results using 20 nearest neighbors were highly consistent with those using 30 neighbors (Supplementary Figure 8b). We selected $k = 30$ in our pipeline for consistency with the scVelo defaults.

ATAC-seq peak files were preprocessed using the standard pipeline from MultiVelo [2], and peaks were annotated to genes using HOMER [13]. The resulting chromatin accessibility values were normalized using the term frequency-inverse document frequency (TF-IDF) method [14]. In addition, cell neighborhoods were constructed based on multi-modality data using the weighted nearest neighbor (WNN) [15] method, and data smoothing was carried out using the 50 neighbors. Finally, spliced and unspliced RNA counts, along with other omics features, were each scaled to the (0, 1) range and concatenated column-wise. Additional modalities can be incorporated following the same preprocessing step: normalization, smoothing, (0, 1) scaling, and column-wise concatenation.

For the other methods, we adhered to standard data processing procedures using default parameters. Monocle3 and Slingshot were provided with cell clusters containing the true starting point. scTour were provided with a predefined direction for pseudotime. For DeepVelo and VeloVI, which do not predict cellular time, we computed pseudotime using the scVelo function *scvelo.tl.velocity_pseudotime*.

7. Computational performance

We systematically evaluated the convergence behavior and computational efficiency of InterVelo. Across representative datasets, we observed that 100 epochs of optimization were sufficient to achieve stable loss convergence (Supplementary Figure 9a).

To evaluate the computational efficiency, we compared the runtime of InterVelo with veloVI, DeepVelo, and scVelo using the pancreas dataset, which includes 3969 cells and 2000 highly variable genes. All computations were performed on the $\boldsymbol{\pi}$ 2.0 cluster supported by the Center for High Performance Computing at Shanghai Jiao Tong University. The system is equipped with dual Intel Xeon Scalable Cascade Lake 6248 CPUs (2.5GHz, 20 cores each) and 192GB of DDR4 ECC registered RAM (12×16GB Samsung modules @ 2666MHz). To ensure a fair comparison, we allocated 10 CPU cores to each method and trained all models for 100 epochs. InterVelo's runtime remains within an acceptable range (Supplementary Figure 9b).

Under the same computational configuration, InterVelo required 1.0 CPU hours to train on the P12+P35 dentate gyrus dataset (2930 cells, 2000 genes), 6.2 CPU hours for P0+P5 dentate gyrus dataset (18213 cells, 2000 genes), 1.0 CPU hours for the metabolic labeled neuron dataset (3060 cells, 2000 genes), and 1.4 CPU hours for the RNA+ATAC cortex dataset (4693 cells, 954 genes, 467316 peaks).

8. Batch size selection

Since the ODE solver operates on mini-batches, the step size is indirectly influenced by the choice of batch size. Our analysis of batch size effects indicates that velocity estimation results show remarkable stability across different batch sizes (Supplementary Figure 8a).

References:

1. Gao M, Qiao C, Huang Y: **UniTVelo: temporally unified RNA velocity reinforces single-cell trajectory inference.** *Nat Commun* 2022, **13:**6586.
2. Li C, Virgilio MC, Collins KL, Welch JD: **Multi-omic single-cell velocity models epigenome-transcriptome interactions and improves cell fate prediction.** *Nat*

Biotechnol 2023, **41:**387-398.

3. Reuter B, Fackeldey K, Weber M: **Generalized Markov modeling of nonreversible molecular kinetics.** *J Chem Phys* 2019, **150:**174103.
4. Weiler P, Lange M, Klein M, Pe'er D, Theis F: **CellRank 2: unified fate mapping in multiview single-cell data.** *Nat Methods* 2024, **21:**1196-1205.
5. Qiao C, Huang Y: **Representation learning of RNA velocity reveals robust cell transitions.** *Proc Natl Acad Sci U S A* 2021, **118**.
6. Bergen V, Lange M, Peidli S, Wolf FA, Theis FJ: **Generalizing RNA velocity to transient cell states through dynamical modeling.** *Nat Biotechnol* 2020, **38:**1408-1414.
7. Cui H, Maan H, Vladoiu MC, Zhang J, Taylor MD, Wang B: **DeepVelo: deep learning extends RNA velocity to multi-lineage systems with cell-specific kinetics.** *Genome Biol* 2024, **25:**27.
8. Wolf FA, Angerer P, Theis FJ: **SCANPY: large-scale single-cell gene expression data analysis.** *Genome Biol* 2018, **19:**15.
9. Wu T, Hu E, Xu S, Chen M, Guo P, Dai Z, Feng T, Zhou L, Tang W, Zhan L, et al: **clusterProfiler 4.0: A universal enrichment tool for interpreting omics data.** *Innovation* 2021, **2:**100141.
10. The Gene Ontology Consortium: **The Gene Ontology Resource: 20 years and still GOing strong.** *Nucleic Acids Res* 2019, **47:**D330-D338.
11. Accogli A, Addour-Boudrahem N, Srour M: **Chapter 4 - Neurogenesis, neuronal migration, and axon guidance.** In *Handbook of Clinical Neurology.* Elsevier; 2020: 25-42
12. Harris CR, Millman KJ, van der Walt SJ, Gommers R, Virtanen P, Cournapeau D, Wieser E, Taylor J, Berg S, Smith NJ, et al: **Array programming with NumPy.** *Nature* 2020, **585:**357-362.
13. Heinz S, Benner C, Spann N, Bertolino E, Lin YC, Laslo P, Cheng JX, Murre C, Singh H, Glass CK: **Simple combinations of lineage-determining transcription factors prime cis-regulatory elements required for macrophage and B cell identities.** *Mol Cell* 2010, **38:**576-589.
14. Zhang K, Hocker JD, Miller M, Hou X, Chiou J, Poirion OB, Qiu Y, Li YE, Gaulton KJ, Wang A, et al: **A single-cell atlas of chromatin accessibility in the human genome.** *Cell* 2021, **184:**5985-6001.e5919.
15. Hao Y, Hao S, Andersen-Nissen E, Mauck WM, 3rd, Zheng S, Butler A, Lee MJ, Wilk AJ, Darby C, Zager M, et al: **Integrated analysis of multimodal single-cell data.** *Cell* 2021, **184:**3573-3587 e3529.

Supplementary Figures.

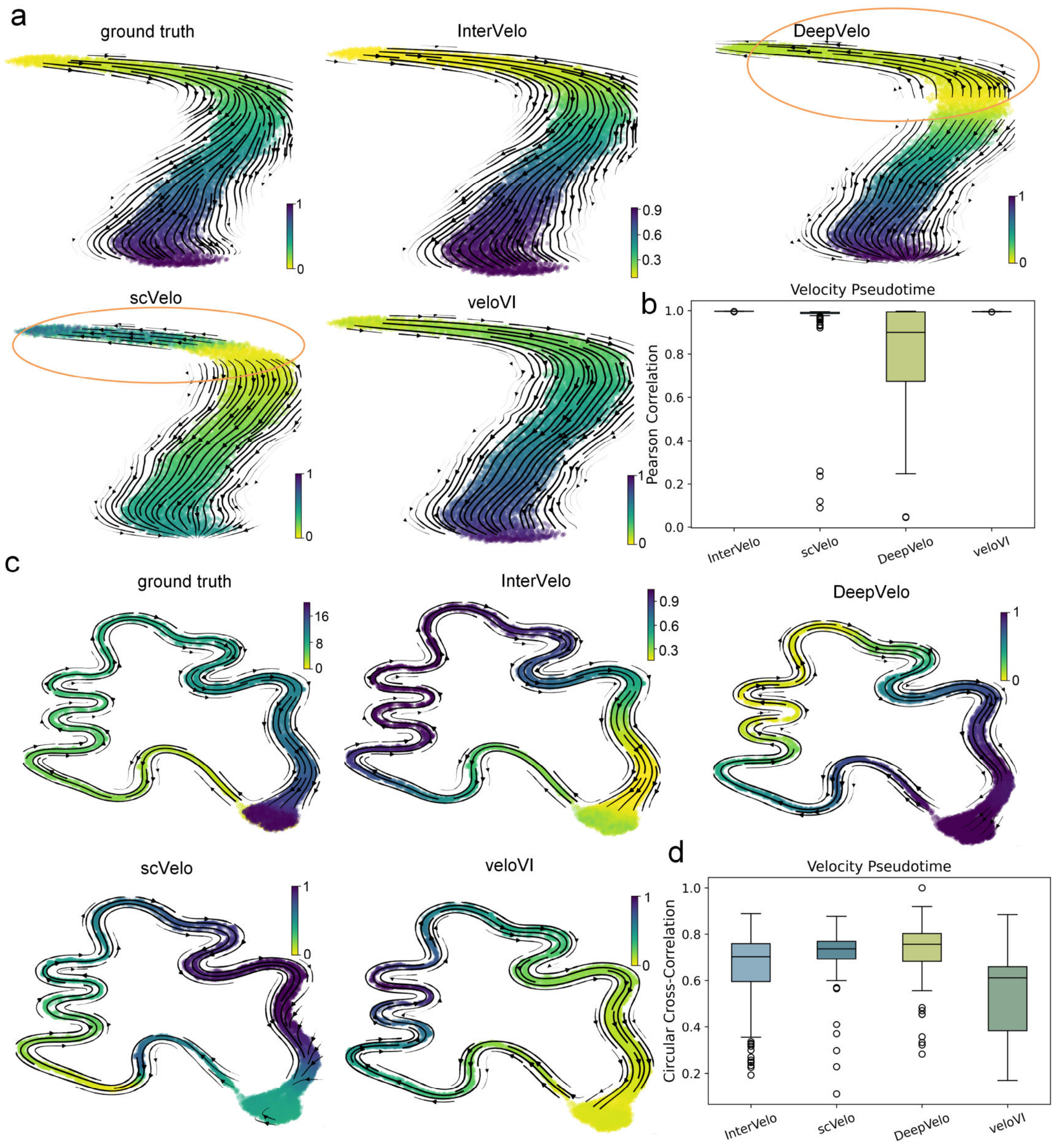

Supplementary Figure 1. Benchmarking of InterVelo in two simulation datasets **a**, **b**, Comparison between InterVelo and other methods in single-omics data with a bell-shaped trajectory. **a**, Streamlines of true velocity and velocity predicted by InterVelo, DeepVelo, scVelo and veloVI are projected to a UMAP embedding, with cells colored according to true time and pseudotime, respectively. **b**, The boxplot of velocity pseudotime Pearson correlation between prediction and ground truth across different methods, evaluated over 100 simulation datasets. **c, d**, Comparison between InterVelo and other methods in multi-omics state-switch data. **c**, Streamlines of true velocity and velocity predicted by InterVelo, DeepVelo, scVelo and veloVI are projected to the

UMAP embedding, with cells colored according to true time and pseudotime, respectively. **d**, The boxplot of velocity pseudotime circular cross-correlation between prediction and ground truth across different methods, evaluated over 100 simulation datasets.

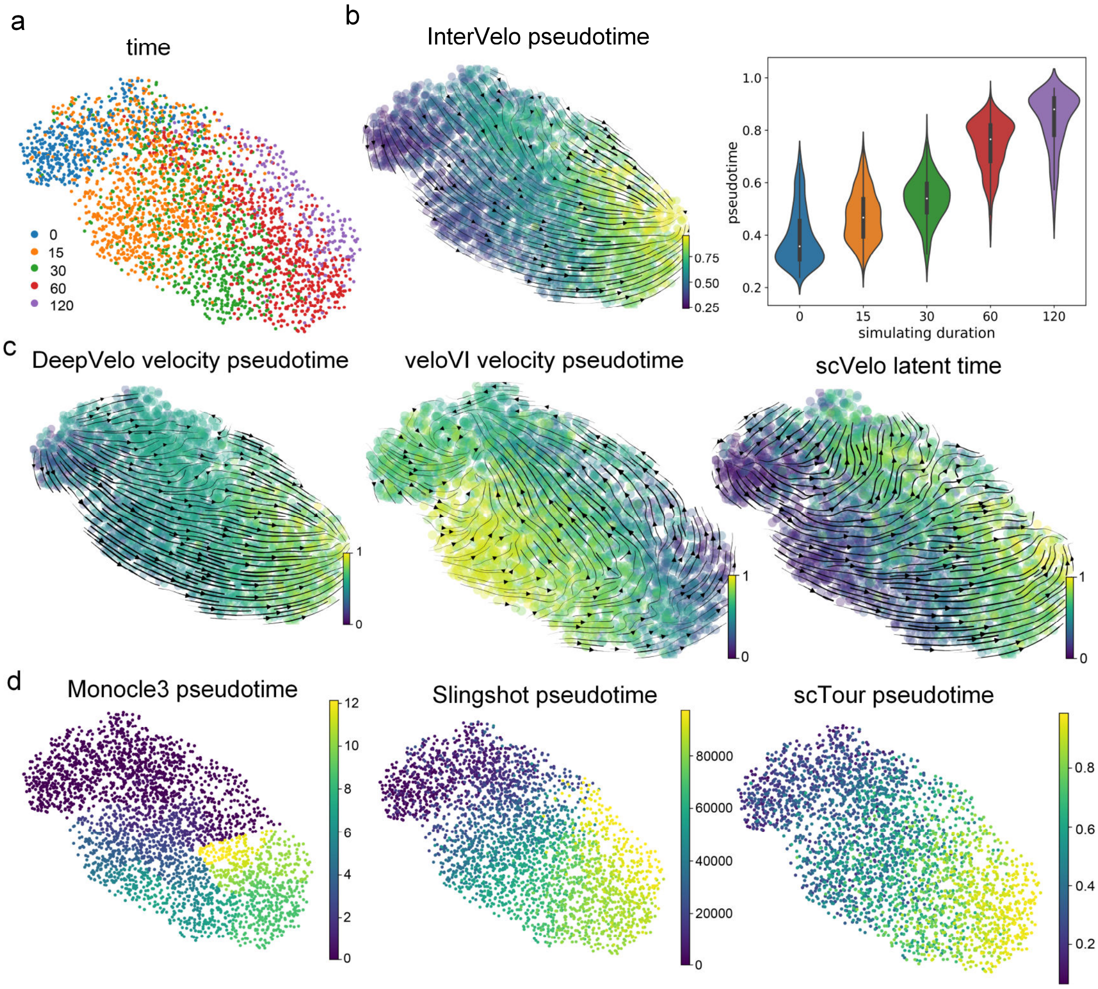

Supplementary Figure 2 Comparison of pseudotime and velocity in metabolic labeled neuron data. **a**, Ground-truth time of the metabolic labeled neuron data. **b**, Velocity field predicted by InterVelo, with cells colored by InterVelo's pseudotime. **c**, Pseudotime and velocity field predicted by DeepVelo, veloVI, and scVelo. Pseudotime for DeepVelo and veloVI was computed using scVelo's function *scvelo.tl.velocity_pseudotime*. **d**, Velocity predictions from Monocle3, Slingshot, and scTour.

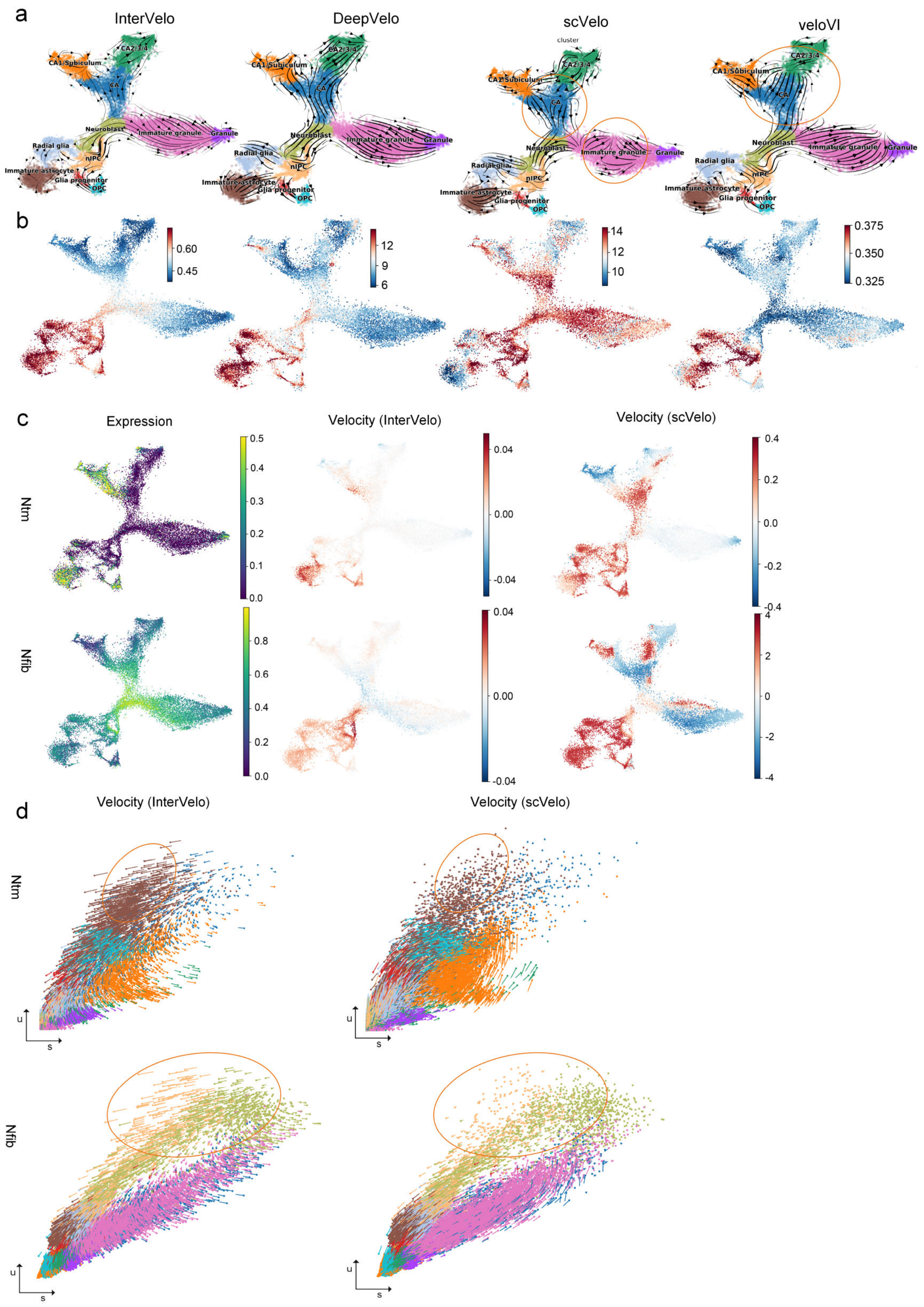

Supplementary Figure 3 InterVelo unravels cellular fates and genetic dynamics in mouse hippocampus development. **a**, **b**, The velocity streamlines (**a**) and velocity length (**b**) derived from InterVelo, scVelo, DeepVelo, veloVI are projected onto t-SNE plots **c**, **d**, Comparison between velocity from InterVelo and scVelo for Ntm and Nfib

genes. **c**, Gene expression and velocities predicted by InterVelo and scVelo for Ntm and Nfib genes are projected onto t-SNE plots. **d**, Scatter plots display the phase portraits of spliced and unspliced RNA for Ntm and Nfib genes, with RNA velocity predicted by InterVelo and scVelo represented as arrows. Colors correspond to cell types, consistent with the color scheme in panel **a**.

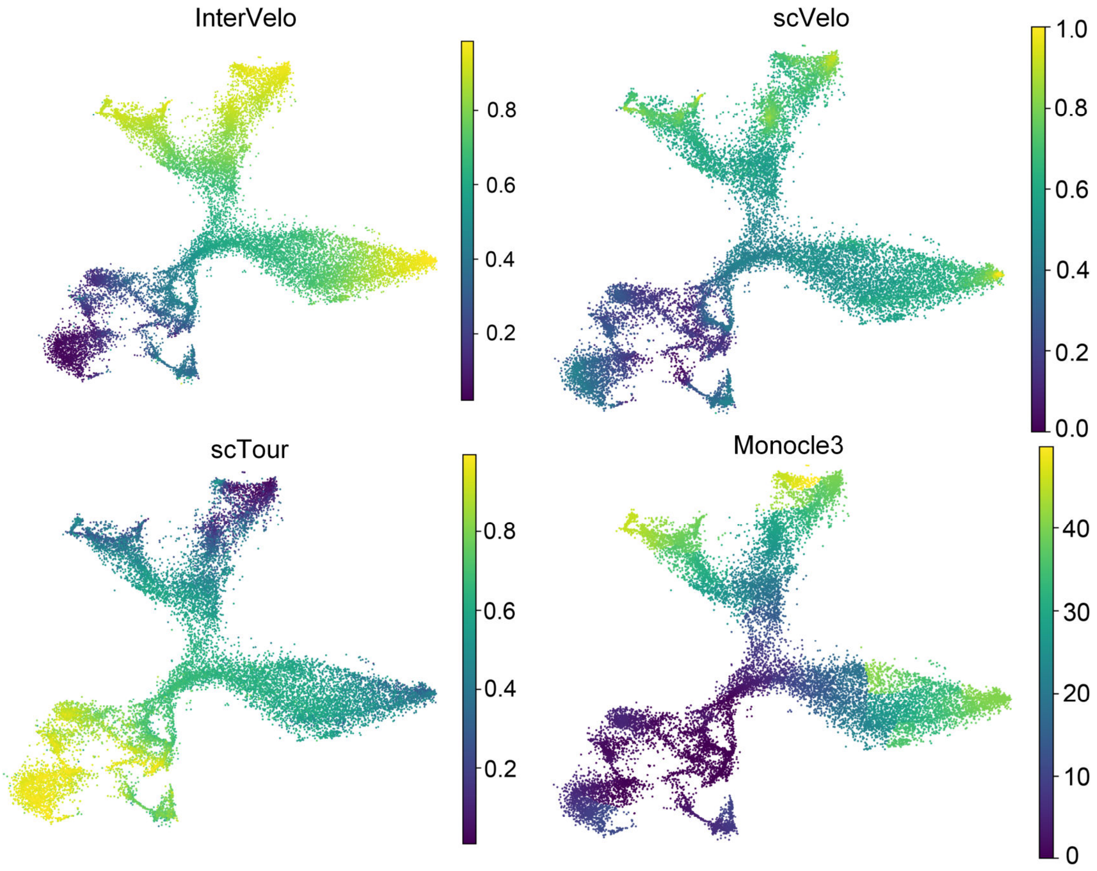

Supplementary Figure 4 The pseudotime predicted using InterVelo, scVelo, scTour and Monocle3 in mouse dentate gyrus development data (P0+P5). Since Slingshot failed to converge, its results were excluded.

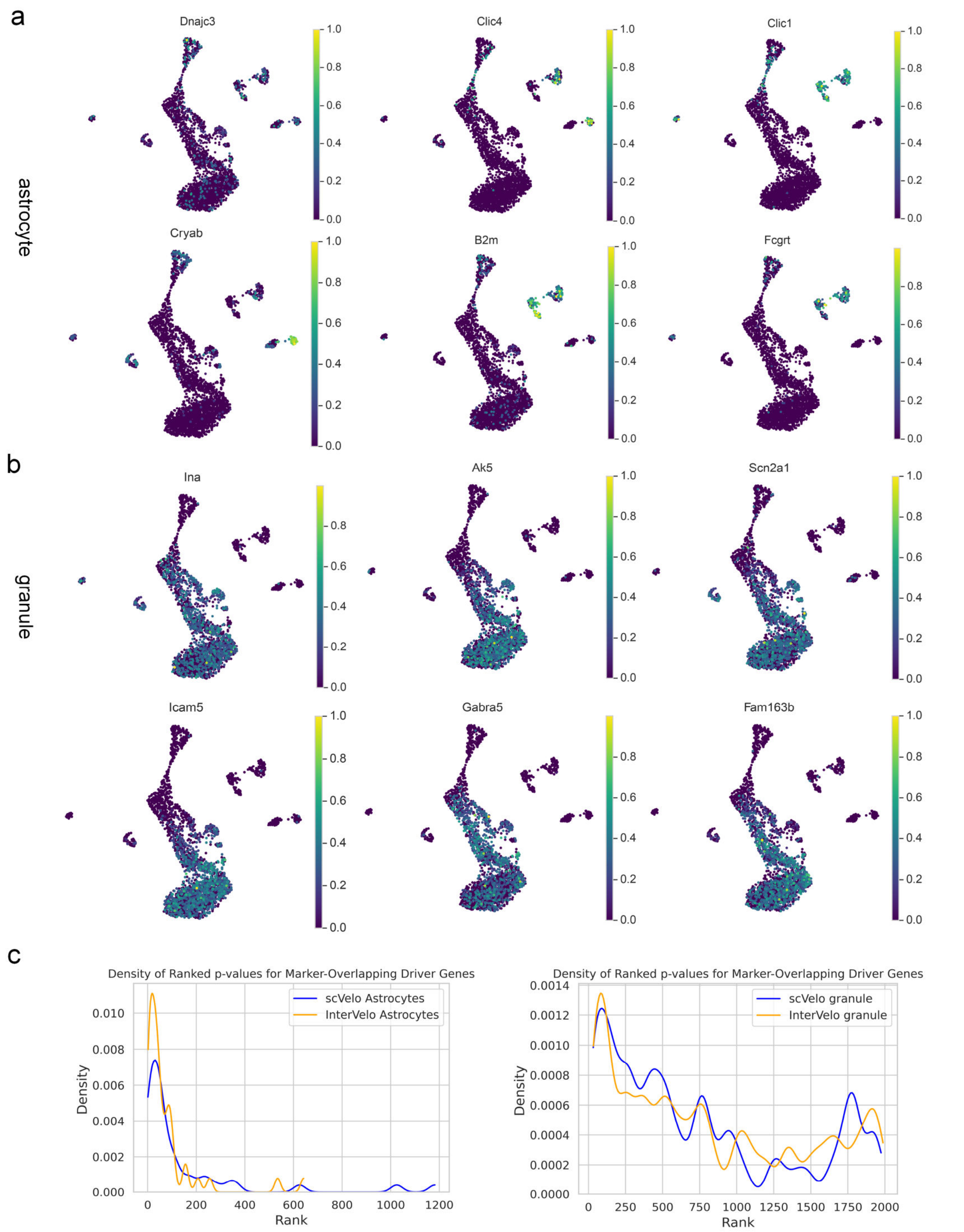

Supplementary Figure 5 InterVelo infers driver genes in the cerebellum **a**, **b**, The expression levels of a part of driver genes inferred by InterVelo, which did not overlap with scVelo, are projected to the UMAP plots. **a**, Driver genes for the astrocyte lineage. **b,** Driver genes for the granule lineage. **c**, The plots show ranking density of marker-overlapping driver genes inferred by scVelo and InterVelo for astrocyte and granule lineages, respectively.

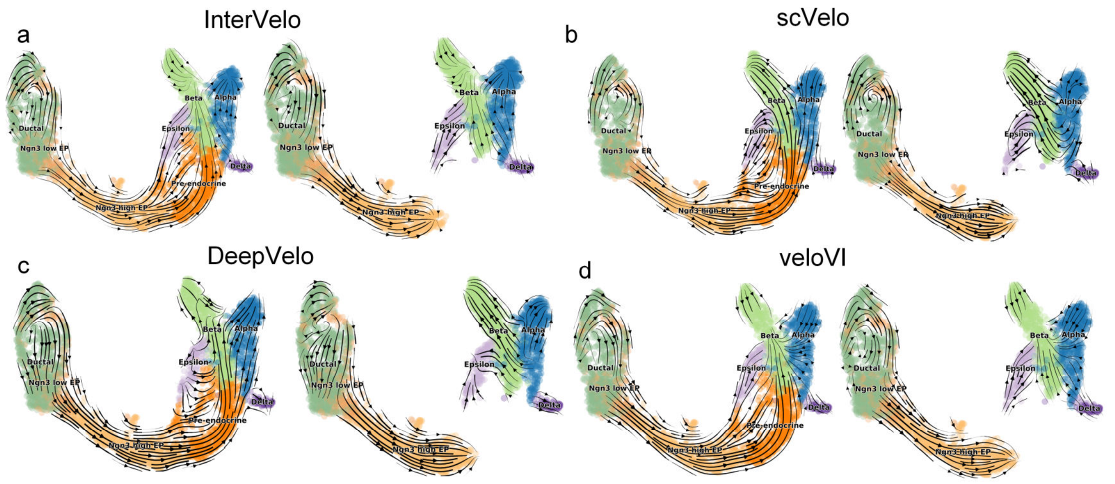

Supplementary Figure 6 The velocity streamlines derived from InterVelo, scVelo, DeepVelo, veloVI in pancreas endocrinogenesis complete dataset and partial dataset are projected to the UMAP plots.

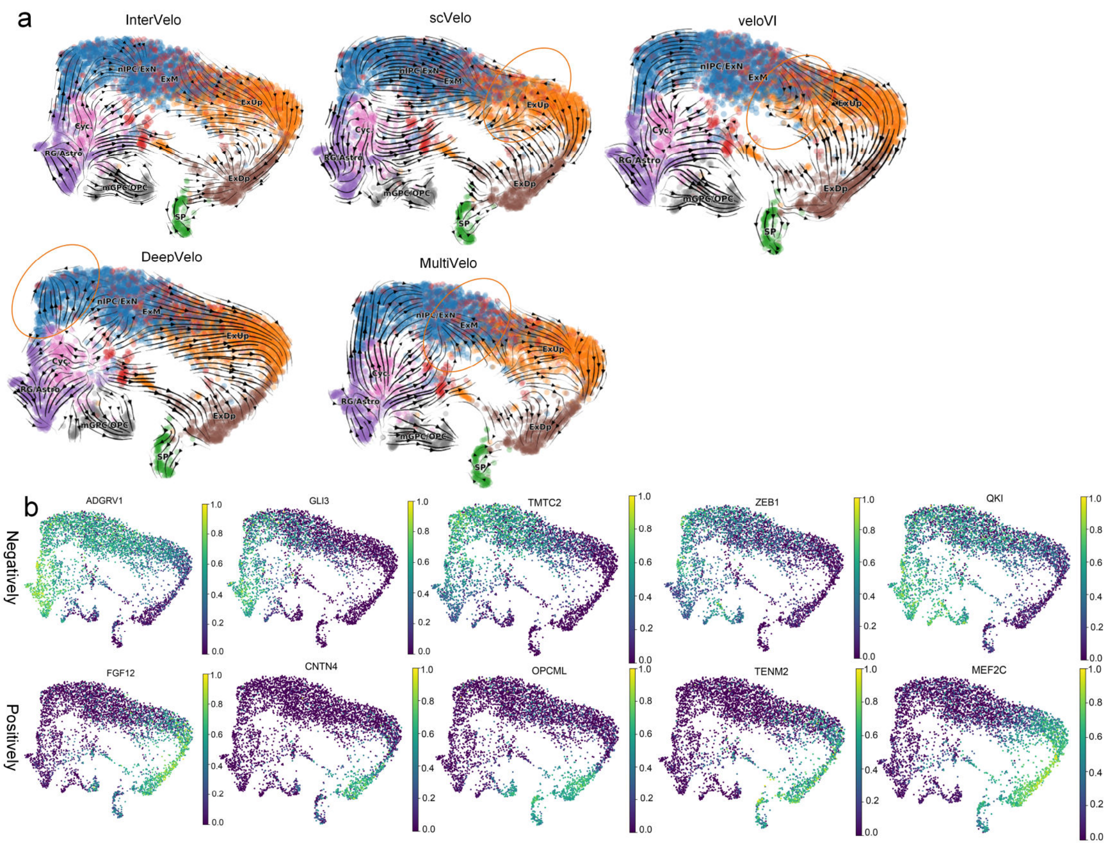

Supplementary Figure 7 **a**, The velocity computed by InterVelo, scVelo, veloVI, DeepVelo and MultiVelo are projected to the UMAP plots respectively. **b**, The UMAP plots show gene expression of top 5 negatively and positively correlated genes along neurogenesis lineage.

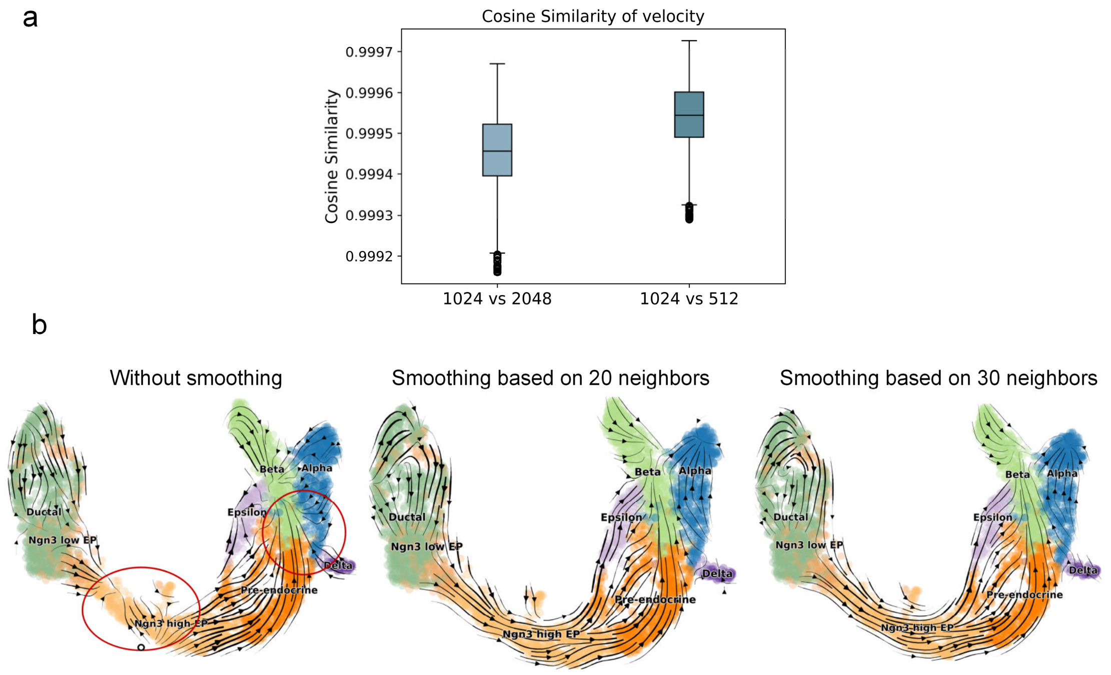

Supplementary Figure 8 Parameter Testing using pancreas data. a, Cosine similarity of pancreas gene velocities estimated using different batch sizes (512 vs 1024 and 1024 vs 2048). b, Velocity estimation in pancreas data under varying smoothing parameters (number of nearest neighbors).

Supplementary Table 1 Four metrics evaluating the accuracy of velocity direction and length in the pancreas dataset under different smoothing parameters.

Methods	CBDir	CBDir2	TransCosine	LenAcc	Mean
Smoothing based on 30 neighbors	0.38	0.22	0.23	0.63	0.41
Smoothing based on 20 neighbors	0.33	0.23	0.24	0.64	0.36
Without smoothing	0.31	0.05	0.05	0.47	0.22

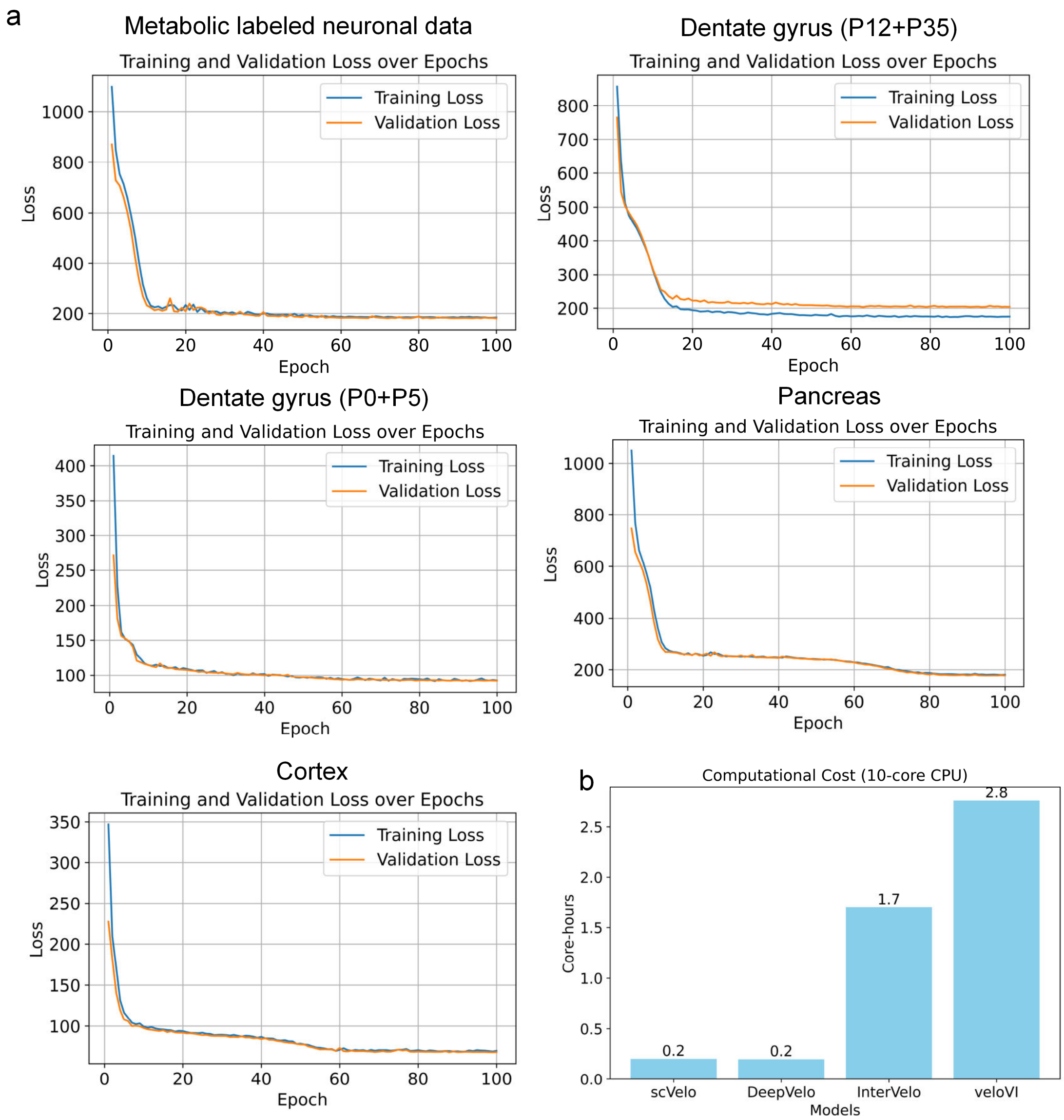

Supplementary Figure 9 Computational performance of InterVelo **a**, Training and validation loss curves of InterVelo across representative datasets over 100 epochs of model training. **b**, Comparison of computational core hours between scVelo, DeepVelo, InterVelo and VeloVI on the pancreas dataset.
